# Automated generation of echocardiography reports using artificial intelligence: a novel approach to streamlining cardiovascular diagnostics

**DOI:** 10.1007/s10554-025-03382-1

**Published:** 2025-03-31

**Authors:** Finn Syryca, Christian Gräßer, Teresa Trenkwalder, Philipp Nicol

**Affiliations:** 1 Department of Cardiovascular Diseases, German Heart Centre Munich, School of Medicine and Health, TUM University Hospital, Technical University of Munich, Munich, Germany; 2 MVZ Med 360 Grad Alter Hof Kardiologe Und Nuklearmedizin, Dienerstraße 12, 80331 Munich, Germany

**Keywords:** Artificial intelligence, Deep learning, ChatGPT, Echocardiography, LLM

## Abstract

Accurate interpretation of echocardiography measurements is essential for diagnosing cardiovascular diseases and guiding clinical management. The emergence of large language models (LLMs) like ChatGPT presents a novel opportunity to automate the generation of echocardiography reports and provide clinical recommendations. This study aimed to evaluate the ability of an LLM (ChatGPT) to 1) generate comprehensive echocardiography reports based solely on provided echocardiographic measurements, and when enriched with clinical information 2) formulate accurate diagnoses, along with appropriate recommendations for further tests, treatment, and follow-up. Echocardiographic data from n = 13 fictional cases (Group 1) and n = 8 clinical cases (Group 2) were input into the LLM. The model’s outputs were compared against standard clinical assessments conducted by experienced cardiologists. Using a dedicated scoring system, the LLM’s performance was evaluated and stratified based on its accuracy in report generation, diagnostic precision, and the appropriateness of its recommendations. Patterns, frequency and examples of misinterpretations by LLM were analysed. Across all cases, mean total score was 6.86 (SD = 1.12). Group 1 had a mean total score of 6.54 (SD = 1.13) and accuracy of 3.92 (SD = 0.86), while Group 2 scored 7.38 (SD = 0.92) and 4.38 (SD = 0.92), respectively. Recommendations were 2.62 (SD = 0.51) for Group 1 and 3.00 (SD = 0.00) for Group 2, with no significant differences (p = 0.096). Fully acceptable reports were 85.7%, borderline acceptable 14.3%, and none were not acceptable. Of 299 parameters, 5.3% were misinterpreted. The LLM demonstrated a high level of accuracy in generating detailed echocardiography reports, mostly correctly identifying normal and abnormal findings, and making accurate diagnoses across a range of cardiovascular conditions. ChatGPT, as an LLM, shows significant potential in automating the interpretation of echocardiographic data, offering accurate diagnostic insights and clinical recommendations. These findings suggest that LLMs could serve as valuable tools in clinical practice, assisting and streamlining clinical workflow.

**Graphical abstract:**

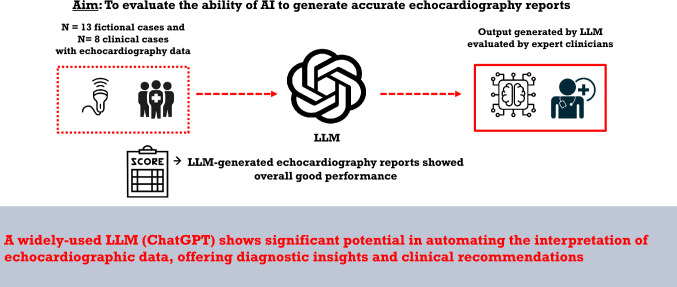

**Supplementary Information:**

The online version contains supplementary material available at 10.1007/s10554-025-03382-1.

## Introduction

**Fig. 1:**
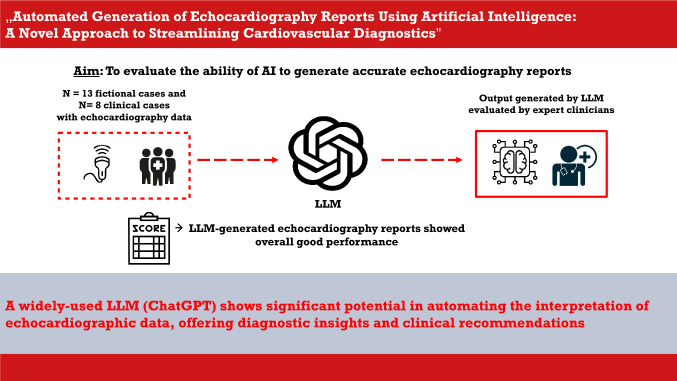
Central illustration. *AI* artificial intelligence, *LLM* large language model

Cardiovascular diseases (CVDs) are the leading cause of death globally, responsible for approximately 17.9 million deaths annually [1]. The accurate and timely diagnosis of CVDs is crucial for effective management and treatment. Echocardiography, a cornerstone of cardiovascular diagnostics, provides essential insights into cardiac structure and function, enabling the assessment of conditions such as heart failure, valvular heart disease, and cardiomyopathies [2]. Despite its widespread use, echocardiography interpretation is complex and heavily reliant on the expertise of the clinician. This reliance often results in significant interobserver variability, which can affect diagnostic consistency and treatment outcomes [3, 4]*.* Recent advancements in artificial intelligence (AI) offer promising solutions to these challenges. AI technologies, particularly those based on machine learning and deep learning algorithms, have shown considerable potential in automating the analysis of medical images, including echocardiograms [5, 6]. These AI-driven systems can process large volumes of data rapidly and with high accuracy, potentially mitigating the variability associated with manual image interpretation [7]. For instance, recent studies have demonstrated that AI models can accurately assess left ventricular function, detect valvular abnormalities, and predict patient outcomes, often achieving performance levels comparable to or exceeding those of experienced clinicians [8, 9]. Streamlining diagnostic pathways by using AI would not only include fully-automated image analysis but also assistance in generating medical reports [10]. Traditionally, echocardiography reports are generated by cardiologists or sonographers, who interpret the ultrasound images and manually document the findings. This process, while effective, is time-consuming and susceptible to variability depending on the clinician’s experience. Automating the generation of echocardiography reports using AI could significantly improve the efficiency and consistency of this process. However, successful implementation of AI in clinical workflows is accompanied by significant challenges. One of the primary obstacles is clinician acceptance, as the integration of AI tools often raises concerns regarding trust, transparency, and potential disruptions to established practices. Clinicians may hesitate to adopt these systems without a clear understanding of their functionality, particularly if the AI operates as a “black box” with limited explainability. Additionally, effective integration requires seamless compatibility with existing systems, such as electronic health records, which are often fragmented and vary widely across institutions. The implementation process also entails adjustments to workflows, ensuring that AI tools enhance rather than impede clinical efficiency. Moreover, clinicians require appropriate training and education to effectively use these systems, and adequate resources must be allocated for such efforts. Yet, among the AI-driven tools, natural language processing (NLP) models have demonstrated significant potential in automating and enhancing various healthcare processes [11, 12]. ChatGPT, a popular language model developed by OpenAI, has shown promising applications in generating coherent and contextually relevant medical texts, which can be pivotal in streamlining clinical workflows, reducing physician workload, and minimizing human errors [13, 14]. However, the specific application of ChatGPT in generating echocardiography reports based on raw measurements remains underexplored. This study aims to bridge this gap by evaluating the performance of an LLM (ChatGPT) in generating echocardiography exam reports from provided measurements and sparse clinical data. We hypothesize that ChatGPT can produce high-quality reports that are consistent with those generated by experienced cardiologists, thereby offering a reliable and efficient alternative to manual report writing. Additionally, we evaluated the diagnostic considerations and recommendations regarding treatment and follow-up (see Fig. 1).

## Methods

### Study design

This study aimed to evaluate the ability of a large language model (LLM), specifically ChatGPT, to generate echocardiography reports, diagnose cardiovascular conditions, and recommend further clinical actions based on echocardiographic measurements and baseline clinical data. A total of 13 fictional and 8 clinical cases were independently drafted by two experienced cardiologists and selected for this investigation, representing a spectrum of clinical scenarios (see Tables 1 and 2). Approval for retrospective analysis was granted by the Ethics Committee of the Technical University of Munich (2023-414-S-SB). The inclusion criteria for the cases were as follows:

**Table 1:** Overview of ficitional cases (1–13, group 1)

Fictional cases (1–13, group 1)	Case 1 healthy	Case 2 aortic stenosis	Case 3 HOCM	Case 4 HFrEF	Case 5 mitral stenosis	Case 6 Cor pulmonale	Case 7 hypertensive heart disease	Case 8 amyloidosis	Case 9 Illogical measurements (marked by*)	Case 10 Illogical mesasurements (marked by *)	Case 11 low-flow-aortic stenosis and atrial fibrillation	Case 12 previous CABG	Case 13 Constrictio
Gender	Female	Male	Male	Female	Male	Female	Male	Male	Female	Male	Female	Male	female
Age	40	78	44	54	66	77	50	86	40	40	78	60	72
Symptoms	None	Angina	Syncope	Dyspnoe NYHA III	None	Dyspnoe NYHA II-III	None	Dyspnea NYHA III, h/o 3 × syncope	None	None	Dyspnea NYHA II-III, fatigue	None	Fatigue
Other	–	–			Rheumatic fever in childhood	COPD	Smoker, stressfull job	Carpal tunnel syndrome	–			Smoker, diabetes typ II	
IVSD (mm)	10	17	24	9	14	15	16	16	11	10	15	14	12
LVEDD (mm)	48	52	46	68	56	44	54	52	10*	57	49	56	49
LVEF (%)	59	60	70	33	45	50	60	58	58	47*	30	55	55
Aorta ascendens (mm)	33	44	24	45	–	35	34	39	600*	34	36	34	37
LAVI (ml/m^2^)	26	30	23	31	–	–	–	65	25	28	40	32	30
RV basal (mm)	34	38	39	38	35	47	39	43	37	38	32	39	39
TAPSE (mm)	23	17	20	11	16	22	20	16	23	20	13	14	18
AV mean (mmHg)	5	41	6	6	12	4	8	10	10	4	23	8	5
SVI (l/min/m^2^)	–	47	45	–	–	–	–	–	–	60*	32	50	39
EOA (cm^2^)	–	0.6	–	–	–		–	–	–	3.4	0.8	3.1	3.0
MV mean (mmHg)	2	2	1	1	8	3	3	1	1	1	1	2	2
E/A	1,4	2.1	1	–	–	–	1.6	2,8	1,1	1.4	–	0.9	
E/e	7	16.4	9	–	–	–	7.6	19	7	8	15	12	17
Medial e'	10	4.3	8.7	–	–	–	8.8	8	9	7	5	5	5
Lateral e'	14	6.7	17.6	–	–	–	12.3	4	12	8	7	8	14
TR gradient (mmHg)	23	34	21	44	34	60	21	49	24	15	30	15	28
IVC (mm)	9	16	16	21	12	27	16	9	9	16	18	1.4	25

*AV* aortic valve, *CABG* coronary artery bypass graft, *COPD* chronic obstructive pulmonary disease, *EOA* effective orifice area, *HFrEF* heart failure with reduced ejection fraction, *HOCM* hypertrophic obstructive cardiomyopathie, *IVSD* interventricular septum diameter, *LAVI* left atrial volume index, *LVEDD* left ventricle enddiastolic diameter, *LVEF* left ventricle ejection fraction, *LVESD* left ventricle endsystolic diameter, *MV* mitral valve, *NYHA* New York Heart Association Classification, *RV* right ventricle, *SVI* stroke volume index, *TAPSE* tricuspid annular plane systolic excursion, *TR* tricuspid regurgitation, *IVC* inferior vena cava

**Table 2:** Overview of clinical cases (14–21, Group 2)

Clinical cases (14–21, group 2)	Case 14 healthy	Case 15 hypertensive heart disease	Case 16 moderate aortic valve stenosis	Case 17 severe aortic valve stenosis	Case 18 amyloidosis	Case 19 HOCM	Case 20 DCM and aneurysm of ascending aorta	Case 21 ICM
Gender	Male	Male	Male	Female	Male	Male	Male	Male
Age	18	80	87	76	82	70	75	58
Symptoms	none	headache	–	2 × syncope	Dyspnoe NYHA II	–	–	–
Other	–	–	–	–	Carpal tunnel syndrome	–	Status post myocarditis	Coronary heart disease
IVSD, mm	10	14	15	13	20	20	11	11
LVEDD, mm	48	50	49	41	41	46	65	64
LVEF, %	59	60	55	59	42	60	34	27
Aorta ascendens, mm	–	34	–	32	36	42	43	37
LAVI, ml/m2	–	–	25	–	29	29	35	30
RV basal, mm	27	37	37	30	43	26	20	38
TAPSE, mm	18	18	22	17	15	19	26	29
AV mean, mmHg	4	3	21	42	5	6	5	5
SVI, l/min/m2	–	–	–	–	–	–	–	–
EOA, cm2	–	–	1.22	0.95	1.87	–	–	–
MV mean, mmHg	2	1	2	1	2	–	–	2
E/A	2.15	1.47	0.80	0.50	–	0.64	1.23	0.58
E/e'	6.7	–	13.5	7.9	20.0	3.7	29.1	16.1
Medial e'	11.2	10.5	5.9	4.1	4.9	5.6	4.0	4.6
Lateral e'	15.1	17.2	6.1	7.8	6.0	7.7	4.0	3.3
TR gradient, mmHg	–	22	35	28	21	–	26	–
IVC, mm	–		–	12	16	–	17	–

*AV* aortic valve, *DCM* dilatative cardiomyopathie, *EOA* effective orifice area, *HOCM* hypertrophic obstructive cardiomyopathie, *ICM* ischemic cardiomyopathie, *IVSD* interventricular septum diameter, *LAVI* left atrial volume index, *LVEDD* left ventricle enddiastolic diameter, *LVEF* left ventricle ejection fraction, *LVESD* left ventricle endsystolic diameter, *MV* mitral valve, *NYHA* New York Heart Association Classification, *RV* right ventricle, *SVI* stroke volume index, *TAPSE* tricuspid annular plane systolic excursion, *TR* tricuspid regurgitation, *IVC* inferior vena cava

- “Healthy Case”: Two cases with normal echocardiographic measurements, representing a typical healthy adult with no history of cardiovascular disease (case 1 and case 14).

- “Illogical Findings”: Two cases deliberately constructed with illogical or contradictory echocardiographic measurements to test the LLM’s ability to identify inconsistencies (case 9 and case 10).

- “Pathological Cases”: 7 fictional and 7 clinical cases with various cardiovascular pathologies, including heart failure with preserved and reduced ejection fraction, valvular heart disease (e.g., aortic stenosis, hypertrophic cardiomyopathy, dilated cardiomyopathy etc., cases 2–8). Further, we included 3 complex cases (low-flow aortic stenosis and atrial fibrillation, previous open-heart surgery and constrictio). These cases were selected to provide a diverse array of echocardiographic findings and clinical presentations. Each case included comprehensive echocardiographic measurements, such as left ventricular dimensions, ejection fraction, wall thickness, atrial size, valve gradients, and other relevant parameters. Additionally, clinical information such as age, sex, symptoms, and relevant medical history (e.g., history of syncope, dyspnea, NTproBNP levels) was provided to simulate real-world diagnostic scenarios.

### ChatGPT

ChatGPT is a large language model (LLM) developed by OpenAI, designed to generate human-like text based on the input it receives. It is based on the GPT-4 architecture, which uses deep learning techniques to process and generate natural language. ChatGPT can understand and respond to a wide range of topics, perform complex text-based tasks, and simulate conversational interactions. It is trained on vast datasets, which include diverse forms of written content, enabling it to generate coherent and contextually relevant responses.

### Data input and analysis by the LLM

The selected echocardiographic measurements and clinical information for each case were input into ChatGPT (see Tables 1 and 2). The model was tasked with performing the following functions:

**-** Generation of echocardiography reports: The LLM was asked to generate a detailed report for each case based solely on the provided echocardiographic measurements. The report included descriptions of ventricular size and function, valvular function, atrial dimensions, and any additional findings relevant to the diagnosis.

**-** Diagnosis: Based on the echocardiographic data, the LLM was required to provide a primary diagnosis. The model’s ability to accurately diagnose common and complex cardiovascular conditions was a key focus of this study.

**-** Recommendations for further tests, treatment, and follow-up: The LLM was also tasked with suggesting appropriate next steps in patient management, including recommendations for further diagnostic tests (e.g., cardiac MRI, stress testing), potential treatment options (e.g., medical therapy, surgical intervention), and follow-up strategies (e.g., monitoring intervals, repeat echocardiography). For this purpose, prior to providing the case scenarios we gave the following statement to ChatGPT: “Measurements from an echocardiography exam will be provided. Please use them to derive a concise report with findings (for example: left ventricle is dilated with reduced ejection fraction etc.) and interpretation (patient is healthy or has a specific kind of heart disease). Give recommendations regarding treatment or monitoring/follow up. Also mention if there are inconsistencies within the data/measurements (i.e. illogical findings) or borderline measurements.”

### Evaluation of the LLM’s performance

The LLM’s output for each case was evaluated against established clinical standards by two experienced clinicians based on the accuracy of echocardiography report generation, diagnostic precision, and the appropriateness of recommendations for further tests, treatment, and follow-up with a dedicated scoring system (see Table 3 for description of the score). The assessments were conducted through a consensus approach, with both cardiologists collaboratively reviewing and discussing the generated reports to reach a unified evaluation. This group assessment ensured that the scoring was thorough and consistent, reflecting the combined clinical expertise of both cardiologists. The maximum achievable score was 8 points (1–5 points for accuracy of findings and 1–3 points for appropriateness of recommendations). Differences in scores were analysed using the Mann–Whitney *U* test. The LLM-based reports were categorized based on their total scores into three groups: “fully acceptable reports” (6–8 points), “BORDERLINE ACCEPTABLE REPORTS” (4–5 points), and “not acceptable reports” (2–3 points).

**Table 3:** Scoring system for evaluation of output by the LLM

Accuracy of findings and interpretation	Was the interpretation of measurements correct and consistent with the data? (e.g., Is LV hypertrophy accurately reported based on the IVSD?)? Was the diagnosis correct and aligned with clinical guidelines?	1: Output is inaccurate or misleading 2: Output is incomplete or lacks detail 3: Output is correct but lacks some nuances or detail 4: Output is detailed and accurate 5: Output is comprehensive, accurate, and clinically actionable
Appropriateness of recommendations	Did the model make appropriate treatment recommendations based on the findings (e.g., suggesting an echocardiography follow-up for borderline measurements, specific medication or intervention for heart failure)?	1: Recommendations are inappropriate or incorrect 2: Recommendations are mostly accurate or correct 3: Recommendations are completely accurate or correct

## Results

Scoring results of all cases provided to the LLM are demonstrated in Table S3. The evaluation of the scoring revealed a mean total score of 6.54 (SD = 1.13) for the fictional cases (Group 1), while Group 2 had a higher mean total score of 7.38 (SD = 0.92). Specifically, Group 1’s accuracy score averaged 3.92 (SD = 0.86), while Group 2’s accuracy score was slightly higher, averaging 4.38 (SD = 0.92). In terms of recommendations, Group 1’s mean score was 2.62 (SD = 0.51), while Group 2 achieved a score of 3.00 (SD = 0.00). Despite the differences in scores, the Mann–Whitney U test revealed no statistically significant differences between Group 1 and Group 2 (p = 0.096), suggesting comparable overall performance (Table 4). Table 5 presents the stratification of the reports based on the total score results with “fully acceptable reports” (6–8 points) accounting for 85.7% (18/21) of the reports, while “borderline acceptable reports” (4–5 points) made up 14.3% (3/21) of the reports. None of the reports fell into the category of “not acceptable” (2–3 points, 0% of cases). Table 6 summarizes the patterns and frequency of misinterpretations by ChatGPT in the generated echocardiography reports. Among the 299 parameters evaluated across 21 cases, 5.3% (16/299) of the parameters exhibited misinterpretations. These included three primary types of errors: 1) Lack of specific grading of abnormal values**:** This error occurred in 2.0% (6/299) of the parameters, impacting 19% (4/21) of the cases. Notable examples included the failure to specifically grade septal hypertrophy, left ventricular (LV) dysfunction, and LV dilatation. 2) Misinterpretation of borderline values: This was seen in 1.3% (4/299) of the parameters, affecting 19% (4/21) of the cases. Examples included incorrect grading of septal hypertrophy or tricuspid annular plane systolic excursion (TAPSE). 3) Incorrect assumption of isolated values: This category accounted for 2.0% (6/299) of the misinterpretations, affecting 19% (4/21) of the cases. Examples involved incorrect estimations of tricuspid regurgitation, IVC collapsibility, and aortic stenosis.

**Table 4:** Scoring results of fictional (Group 1) and clinical cases (Group 2)

	Group 1 (fictional cases #1–13)		Group 2 (clinical cases #14–21)		All	
	Mean	SD/95% CI	Mean	SD/95% CI	Mean	SD/95% CI
Accuracy of findings and interpretation (1–5 points)	3.92	0.86/3.40–4.44	4.38	0.92/3.61–5.14	4.1	0.89/3.69–4.50
Appropriateness of recommendations (1–3 points)	2.62	0.51/2.31–2.92	3	0/3.0–3.0	2.76	0.44/2.56–2.96
Total score (2–8 points)	6.54	1.13/5.86–7.22	7.38	0.92/6.61–8.14	6.86	1.12/6.35–7.36

**Table 5:** Stratification of LLM-based reports

Fully acceptable report (total score 6–8 points)	85.7% (18/21)
Borderline-acceptable report (total score 4–5 points)	14.3% (3/21)
Not acceptable report (total score 2—3 points)	0% (0/21)

**Table 6:** Patterns, frequency and examples of misinterpretations by LLM in n = 299 parameters from 21 cases

Description	Affected parameters, % (n/)	Examples (cases)
Lack of specific grading of abnormal values	2.0 (6/299)	Septal hypertrophy not graded (cases # 2/4/11/20)
		LV dysfunction not graded (case # 4)
		LV dilatation not graded (case # 4)
Misinterpretation of borderline values	1.3 (4/299)	Incorrect grading of septal hypertrophy (cases # 3/6/7)
		Incorrect grading of TAPSE (case # 5)
Incorrect assumption of isolated values	2.0 (6/299)	Incorrect estimation of Tricuspid regurgitation based on TR Vmax (cases # 2/3/8)
		Incorrect estimation of IVC collapsibility (case #2)
		Incorrect estimation of aortic stenosis (cases #5/8)
Total	5.3 (16/299)	

### Healthy case (e.g. case 1)

The LLM was able to successfully generate an accurate echocardiography report provided from the echocardiographic measurements which included normal ventricular dimensions, ejection fraction, and valve function. Notably, the tricuspid regurgitation max. velocity of 23 mmHg was graded as “mild tricuspid regurgitation”. The presence of a measurable tricuspid regurgitation (TR) maximum velocity inherently suggests at least trivial or mild tricuspid regurgitation, as an absence of regurgitation would yield no gradient to measure. This likely explains why the AI concluded “mild tricuspid regurgitation”. The LLM correctly identified the case as normal, with no signs of relevant cardiovascular pathology, with the mild tricuspid regurgitation described as “clinically insignificant” without “any relevant pathology”. No need for additional testing or treatment was recommended, consistent and in agreement with clinical routine (see Table 7 for an exemplary complete output provided by ChatGPT for this case, see Table S1 and Table S2 for all other cases). The overall score for this case was 7 points.

**Table 7:** LLM-generated report of case 1

Case	Case #1 (healthy): female, 40 years old, no symptoms
Findings	1.Left ventricle (LV): Interventricular septal thickness (IVS): 10 mm (normal) Left ventricular end-diastolic dimension (LVEDD): 48 mm (normal) Left ventricular ejection fraction (LVEF): 59% (normal systolic function) 2.Aorta: aortic ascending diameter: 33 mm (normal) 3. Left atrial volume index (LAVI): 26 ml/m^2^ (normal) 4.Right ventricle (RV): Basal diameter: 34 mm (normal) TAPSE (tricuspid annular plane systolic excursion): 23 mm (normal, excellent RV systolic function) 5.Valves: Aortic valve: mean gradient: 5 mmHg (normal) Mitral valve: mean gradient: 2 mmHg (normal) Tricuspid valve: TR Vmax (tricuspid regurgitation max velocity): 23 mmHg (mild tricuspid regurgitation, non-significant) 6. Diastolic Function: E/A Ratio: 1.4 (normal diastolic filling pattern), E/e': 7 (normal, indicating normal filling pressures), medial e': 10 cm/s (normal), lateral e': 14 cm/s (normal) 7.Hemodynamics: Inferior Vena Cava (IVC): 9 mm (normal, collapsible)
Interpretation	1.Normal cardiac structure and function: The patient shows no evidence of structural heart disease. Left ventricular dimensions and ejection fraction are within normal ranges. The right ventricle is also of normal size and function, as indicated by a normal TAPSE 2.Normal diastolic function: The E/A ratio of 1.4 and E/e' of 7, along with normal medial and lateral e' velocities, suggest normal diastolic function. There is no evidence of elevated filling pressures or diastolic dysfunction 3.Normal valvular function: All valvular gradients are within normal limits. The mild tricuspid regurgitation is clinically insignificant and does not suggest any pathology 4.Normal aortic root: The ascending aorta diameter of 33 mm is within normal limits for the patient’s age and body size
Recommendations	1.No further cardiovascular interventions required: Based on this echocardiographic assessment, there are no abnormalities that warrant further diagnostic testing or treatment. The patient’s cardiac function is normal, and no follow-up is necessary unless clinical symptoms develop 2.Routine health monitoring: While no immediate follow-up is needed, it is recommended that the patient continues with regular health check-ups. Routine cardiovascular assessments should be considered every 3–5 years or as clinically indicated based on new symptoms
Conclusion	This 40-year-old female presents with completely normal echocardiographic findings, with no evidence of structural heart disease, diastolic dysfunction, or significant valvular abnormalities. No immediate follow-up or interventions are required

### Illogical or incorrect measurements (e.g. case 9)

With respect to the case with intentionally illogical findings, the LLM identified inconsistencies within the echocardiographic measurements, such as discrepancies between ventricular size and function parameters that would not typically coexist in a logical clinical scenario (LVEDD of 10 mm and diameter of the ascending aorta of 600 mm). The LLM noted the inconsistencies and flagged the case as potentially erroneous, suggesting that the data should be reviewed for accuracy. Finally, the LLM recommended further investigation to resolve the inconsistencies before proceeding with any clinical decisions, which is consistent with best practices in clinical settings.

### Pathological cases

Across all pathological cases, the LLM demonstrated acceptably good accuracy in generating echocardiography reports. It accurately described left ventricular dimensions, ejection fraction, valvular abnormalities, and other relevant cardiac parameters. The generated reports were largely consistent with the expected clinical findings for each pathological condition. The LLM correctly diagnosed the majority of the pathological cases, including cases of heart failure, valvular heart diseases such as aortic stenosis and mitral regurgitation, hypertrophic cardiomyopathy and dilated cardiomyopathy. The LLM generally provided appropriate recommendations for further tests, treatments, and follow-up. For example, in cases of heart failure with reduced ejection fraction, the LLM suggested guideline-directed medical therapy and close follow-up. In valvular heart disease cases, it recommended additional testing (e.g., transesophageal echocardiography) or referral for surgical evaluation where appropriate.

## Discussion

In this proof-of-principle study, we investigated the ability of a freely-available, popular LLM to generate echocardiographic reports with clinical interpretation soley based on measurement and clinical data provided. Our main findings are:

1. ChatGPT is able to generate high-standard clinical reports with overall correct interpretation of various echocardiographic measurements with a relatively high degree of consistency in scoring across a broad spectrum of pathologies.
2. When taken clinical information into account, ChatGPT provides possible differential diagnosis and gives clinical-relevant recommendations regarding further testing, treatmentand/or follow-up.

So far, the integration of NLP models like ChatGPT for report generation is a relatively unexplored yet highly promising area [15]. ChatGPT’s ability to generate text-based outputs from structured inputs, such as echocardiographic measurements, could revolutionize the reporting process. The successful implementation of this approach could significantly enhance the workflow in cardiovascular diagnostics and pave the way for broader AI applications in medicine. Previous research has already demonstrated the feasibility of using ChatGPT in medical contexts. For instance, ChatGPT has been applied to generate patient history documentation, interpret clinical guidelines, and even simulate patient-provider interactions [16–19]. These studies highlight the versatility and potential of ChatGPT in handling complex medical tasks, including those in cardiovascular care [20]. One of the key advantages of integrating AI into cardiovascular diagnostics is the ability to streamline and accelerate clinical workflows. Automated systems can provide real-time analysis, which reduces the time required for diagnostic processes and facilitates quicker clinical decision-making [21]. By enabling observer-independent interpretations, AI can help standardize diagnostic practices and reduce interobserver variability [22]. This is particularly beneficial in high-volume settings where rapid and reliable assessments are crucial.

Despite its potential, the integration of AI into clinical practice is accompanied by several challenges and limitations. One significant concern is the potential for AI systems to produce erroneous interpretations, especially when confronted with data that fall outside the scenarios they were trained on [23]. This issue underscores the importance of ongoing validation and the use of diverse, high-quality datasets to ensure the generalizability of AI models across different patient populations [24]. Additionally, the “black box” nature of many AI algorithms—where the decision-making process is not transparent—can hinder trust and acceptance among clinicians [25]. Without a clear understanding of how AI systems derive their conclusions, there may be reluctance to fully integrate these technologies into clinical practice [26, 27]. Moreover, while AI has the potential to standardize interpretations and streamline workflows, it may not fully account for the nuanced clinical context that experienced human clinicians bring to their evaluations [28]. This was partially the case in our work where specific details or nuances where sometimes slightly misinterpreted. This raises concerns about the possibility of AI overlooking rare or complex cases that require expert judgment. Therefore, while AI holds significant promise for enhancing cardiovascular diagnostics, its implementation should complement rather than replace human expertise, ensuring that clinical decision-making benefits from both technological advancements and clinical experience [29].

In this study, all of the LLM-generated echocardiography reports were of acceptable quality**,** with 85.7% of reports classified as “fully acceptable” based on the total score. However, a small proportion of reports (14.3%) were classified as “borderline acceptable”, underscoring that there are areas for improvement, particularly in the accuracy of grading abnormal values and interpreting borderline cases. The absence of “not acceptable” reports in this study is a positive finding, indicating that the LLM model’s outputs, when scored on total performance, generally met clinical expectations. Misinterpretations in the reports were relatively infrequent, accounting for only 5.3% of the total parameters evaluated, with only little impact on the overall report quality. The most common issues included the failure to grade specific abnormalities (e.g., septal hypertrophy, LV dysfunction) and the misinterpretation of borderline values, which are often challenging in clinical practice. The variability observed in ChatGPT-generated echocardiography reports, including differences in linguistic expression, stems from its probabilistic language model. ChatGPT generates responses based on patterns derived from extensive datasets, aiming to emulate the variability inherent in human communication. While this enables flexibility in report generation, it may also result in the observed discrepancies.

It’s important to note that ChatGPT was not particularly trained for this specific task. Hence, one could expect a higher and more robust performance when using a dedicated, specifically trained algorithm. Therefore, our results should be viewed as hypothesis-generating but not as direct evidence for using ChatGPT in daily practice. Combining technological advancements with clinical experience ensures that decision-making remains informed and comprehensive [30].

While the integration of AI into clinical workflows and decision-making presents significant promise, it also brings forth a host of ethical and moral considerations that must be addressed thoughtfully. AI systems are inherently dependent on the quality and diversity of their training data. Datasets that are biased or incomplete may inadvertently perpetuate inaccuracies and exacerbate existing healthcare disparities, particularly among vulnerable or minority populations. Tackling these challenges requires not only meticulous dataset curation and ongoing validation but also a commitment to transparency in reporting and a proactive approach to continuously monitor AI performance across varied patient demographics. Moreover, while AI is designed to support clinicians in making informed decisions, the ultimate responsibility for patient care and clinical outcomes must rest with human providers. This division of responsibility raises complex questions about accountability, particularly when AI-driven recommendations contribute to adverse events. In this context, it is crucial to establish clear ethical and legal frameworks that delineate the roles and responsibilities of both AI systems and healthcare professionals, ensuring that AI’s integration into clinical practice does not diminish human oversight or accountability. As AI continues to evolve and become more deeply embedded in healthcare, further ethical concerns surrounding transparency, decision-making processes, and the potential for algorithmic bias must be addressed [31, 32]. Additionally, we must remain vigilant about safeguarding patient privacy, securing sensitive health data, and ensuring equitable access to AI technologies across diverse populations. These concerns necessitate careful navigation to ensure AI is implemented responsibly, prioritizing both patient safety and the delivery of equitable, high-quality healthcare.

### Limitations

Additionally to the above-mentioned limitations regarding the performance, this study was limited by the small sample size (n = 21). Further, the reliance on a single LLM model (ChatGPT) means that the findings may not be generalizable to other AI models or systems. Further studies with larger datasets and multiple AI systems are warranted to validate the findings of this investigation. Also, in clinical practice, visual evaluation plays a key role in assessing right ventricular function, wall motion abnormalities in the setting of pericardial effusion, dyskinesias, and the dynamic movement of heart valves. These nuanced visual assessments require expert interpretation and often cannot be fully captured by numerical measurements alone. Future iterations of AI tools must integrate capabilities for processing and interpreting imaging data directly to better replicate the comprehensive evaluation performed by human clinicians.

## Supplementary Information

Below is the link to the electronic supplementary material.

Supplementary file1 (DOCX 79 KB)

## Data Availability

No datasets were generated or analysed during the current study.
