# Supplementary material for: Automated generation of echocardiography reports using artificial intelligence: a novel approach to streamlining cardiovascular diagnostics
Source: Int J Cardiovasc Imaging. 2025 Mar 31;41(5):967–77. doi: 10.1007/s10554-025-03382-1 (PMC12075404; doi:10.1007/s10554-025-03382-1)
Supplement: Supplementary file 1 — Supplementary file1 (DOCX 79 KB) [file 10554_2025_3382_MOESM1_ESM.docx]

| **Case** | **Case #2 (Aortic stenosis): male, 78 years old, angina** |
| --- | --- |
| **Findings** | 1. **Left Ventricle (LV):**   **Interventricular Septal Thickness (IVSD):** 17 mm (increased, indicating left ventricular hypertrophy, possibly secondary to aortic stenosis)  **Left Ventricular End-Diastolic Dimension (LVEDD):** 52 mm (normal)  **Left Ventricular Ejection Fraction (LVEF):** 60% (normal systolic function)   1. **Aorta: Aortic Ascending Diameter:** 44 mm (mild dilation) 2. **Left Atrial Volume Index (LAVI):** 30 ml/m² (normal) 3. **Right Ventricle (RV):**   **Basal Diameter:** 38 mm (normal)  **TAPSE (Tricuspid Annular Plane Systolic Excursion):** 17 mm (normal RV systolic function)   1. **Valves:**   **Aortic Valve:** **Mean Gradient:** 41 mmHg (indicative of severe aortic stenosis), **Effective Orifice Area (EOA):** 0.6 cm² (severe aortic stenosis)  **Mitral Valve:** **Mean Gradient:** 2 mmHg (normal)  **Tricuspid Valve:** **TR Vmax (Tricuspid Regurgitation Max Velocity):** 34 m/s (mild tricuspid regurgitation)   1. **Diastolic Function:**   **E/A Ratio:** 2.1 (indicative of Grade II diastolic dysfunction), **E/e’:** 16.4 (elevated, suggesting increased filling pressures), **Medial e’:** 4.3 cm/s (reduced, indicating impaired relaxation), **Lateral e’:** 6.7 cm/s (reduced, consistent with diastolic dysfunction)   1. **Hemodynamics:**   **Stroke Volume Index (SVI):** 47 ml/m² (normal)  **Inferior Vena Cava (IVC):** 16 mm (normal, collapses adequately with inspiration) |
| **Interpretation** | 1. **Severe Aortic Stenosis:**   The patient has a significantly elevated aortic valve mean gradient (41 mmHg) and a critically reduced effective orifice area (0.6 cm²), which confirms severe aortic stenosis. This explains the patient's angina, as severe aortic stenosis can lead to increased myocardial oxygen demand and decreased coronary perfusion.   1. **Left Ventricular Hypertrophy (LVH):**   The increased interventricular septal thickness (17 mm) is indicative of left ventricular hypertrophy, likely secondary to the chronic pressure overload from severe aortic stenosis.   1. **Diastolic Dysfunction (Grade II):**   The elevated E/e’ (16.4), combined with reduced medial and lateral e’ velocities, points to impaired left ventricular relaxation and increased filling pressures, consistent with Grade II (moderate) diastolic dysfunction.   1. **Mild Aortic Root Dilation:**   The ascending aorta measures 44 mm, which is mildly dilated and should be monitored for potential progression.   1. **Normal Right Ventricular Function:**   The right ventricular dimensions (RV basal diameter: 38 mm) and systolic function (TAPSE: 17 mm) are within normal limits.   1. **Trivial Tricuspid Regurgitation:**   The mild tricuspid regurgitation (TR Vmax: 34 m/s) is clinically insignificant and does not require intervention. |
| **Recommendations** | 1. **Aortic Valve Intervention:**   The patient requires urgent evaluation for **aortic valve replacement** (AVR) due to severe aortic stenosis. Both **surgical AVR** and **transcatheter aortic valve replacement** (TAVR) should be considered, with the choice depending on the patient’s surgical risk and overall health status.   1. **Management of Angina:**   While AVR is the definitive treatment, the patient’s angina can be managed in the interim with medications like beta-blockers or nitrates, although caution is required as some medications may worsen the hemodynamics in severe aortic stenosis.   1. **Coronary Angiography:**   Given the patient’s angina and age, a **coronary angiogram** should be performed to rule out concomitant coronary artery disease, which can coexist with aortic stenosis and may require additional intervention (e.g., coronary revascularization).   1. **Monitoring of Aortic Root:**   Serial imaging (e.g., echocardiography or CT scan) should be performed to monitor the size of the ascending aorta, given the mild dilation.   1. **Follow-Up:**   Close follow-up with cardiology is essential to assess the patient’s clinical status and determine the timing of AVR. Regular echocardiograms (every 6-12 months) are recommended if AVR is not performed immediately. |
| **Conclusion** | This 78-year-old male presents with severe aortic stenosis, left ventricular hypertrophy, and diastolic dysfunction, all of which are contributing to his symptoms of angina. Aortic valve replacement is strongly recommended to alleviate symptoms and prevent further cardiovascular deterioration. |

| **Case** | **Case #3 (HOCM): male, 44 years old, syncope** |
| --- | --- |
| **Findings** | 1. **Left Ventricle (LV):**   **Interventricular Septal Thickness (IVSD):** 24 mm (marked left ventricular hypertrophy)  **Left Ventricular End-Diastolic Dimension (LVEDD):** 46 mm (normal)  **Left Ventricular Ejection Fraction (LVEF):** 70% (hyperdynamic systolic function)   1. **Aorta: Aortic Ascending Diameter:** 24 mm (normal) 2. **Left Atrial Volume Index (LAVI):** 23 ml/m² (normal) 3. **Right Ventricle (RV):**   **Basal Diameter:** 39 mm (normal)  **TAPSE (Tricuspid Annular Plane Systolic Excursion):** 20 mm (normal RV systolic function)   1. **Valves:**   **Aortic Valve:** **mean Gradient:** 6 mmHg (normal)  **Mitral Valve:** **mean Gradient:** 1 mmHg (normal)  **Tricuspid Valve:** **TR Vmax (Tricuspid Regurgitation Max Velocity):** 21 m/s (no significant tricuspid regurgitation)   1. **Diastolic Function:**   **E/A Ratio:** 1 (normal filling pattern), **E/e’:** 9 (normal, no evidence of elevated filling pressures), **Medial e’:** 8.7 cm/s (normal), **Lateral e’:** 17.6 cm/s (enhanced relaxation, normal)   1. **Hemodynamics:**   **Stroke Volume Index (SVI):** 45 ml/m² (normal)  **Inferior Vena Cava (IVC):** 16 mm (normal) |
| **Interpretation** | 1. **Severe Left Ventricular Hypertrophy (LVH):**   The markedly increased interventricular septal thickness (24 mm) suggests **severe left ventricular hypertrophy**. Given the patient’s age and lack of significant systemic hypertension, this finding raises suspicion for **hypertrophic cardiomyopathy (HCM)**, a condition characterized by thickened myocardium, often associated with syncope and increased risk of sudden cardiac events.   1. **Normal Systolic and Diastolic Function:**   The left ventricular systolic function is hyperdynamic (LVEF: 70%), which is typical in hypertrophic cardiomyopathy. Diastolic parameters (E/A ratio of 1, E/e’ of 9) suggest preserved diastolic function at this stage.   1. **Normal Right Ventricular Function:**   Right ventricular size and function are within normal limits, as indicated by the TAPSE (20 mm) and RV basal diameter (39 mm).   1. **No Significant Valve Disease:**   The aortic and mitral valves show no evidence of significant stenosis or regurgitation.   1. **Syncope and Risk of Sudden Cardiac Events:**   The presence of syncope in this patient is concerning in the context of severe LVH, particularly in suspected hypertrophic cardiomyopathy, as it may indicate a higher risk of arrhythmias or sudden cardiac death. |
| **Recommendations** | 1. **Further Evaluation for Hypertrophic Cardiomyopathy (HCM):**   This patient’s severe left ventricular hypertrophy with a history of syncope is highly suggestive of hypertrophic cardiomyopathy. **Genetic testing** and **family screening** are recommended if HCM is confirmed. Additionally, a **cardiac MRI** can be useful to further characterize the extent and distribution of myocardial hypertrophy.   1. **Risk Stratification for Sudden Cardiac Death:**   Given the history of syncope and the significant hypertrophy, the patient is at **high risk for sudden cardiac death (SCD)**. Evaluation for the placement of an **implantable cardioverter-defibrillator (ICD)** should be considered for primary prevention.   1. **Holter Monitoring:**   A **24 - 48 hours Holter monitor** or event monitor is recommended to detect any underlying arrhythmias, especially **ventricular tachyarrhythmias**, which may explain the syncope and increase the risk for SCD.   1. **Lifestyle Modifications and Activity Recommendations:**   The patient should be advised to avoid **strenuous physical activity** or competitive sports, as these can increase the risk of arrhythmias in hypertrophic cardiomyopathy.   1. **Follow-Up:**   Regular cardiology follow-up is essential to monitor the progression of hypertrophy, symptoms, and arrhythmic risk. Serial echocardiograms should be performed every 6-12 months or earlier if symptoms worsen. |
| **Conclusion** | This 44-year-old male presents with severe left ventricular hypertrophy, likely due to hypertrophic cardiomyopathy, in the context of syncope. Given the high risk of sudden cardiac death, further diagnostic testing and consideration for ICD implantation are recommended. Close monitoring and lifestyle adjustments are critical for long-term management. |

| **Case** | **Case #5 (HFrEF): female, 54 years old, dyspnoe NYHA III** |
| --- | --- |
| **Findings** | 1. Left Ventricle (LV):   IVSD (Interventricular Septal Thickness): 9 mm (normal)  LVEDD (Left Ventricular End-Diastolic Dimension): 68 mm (dilated)  LVEF (Left Ventricular Ejection Fraction): 33% (reduced, indicating systolic dysfunction)   1. Aorta: Ascending Aorta Diameter: 45 mm (mildly dilated) 2. Left Atrium: LAVI (Left Atrial Volume Index): 31 ml/m² (normal) 3. Right Ventricle (RV):   Basal Diameter: 38 mm (normal)  TAPSE (Tricuspid Annular Plane Systolic Excursion): 11 mm (reduced, indicating RV systolic dysfunction)   1. Valves:   Aortic Valve (AV): Mean gradient 6 mmHg (no significant aortic stenosis)  Mitral Valve (MV): Mean gradient 1 mmHg (normal)  Tricuspid Regurgitation (TR): TR Vmax 44 cm/s, consistent with mildly elevated pulmonary pressures   1. Hemodynamics:   Inferior Vena Cava (IVC): Diameter 21 mm |
| **Interpretation** | This 54-year-old female with NYHA III symptoms presents with dilated cardiomyopathy (DCM) as evidenced by a significantly dilated left ventricle (LVEDD: 68 mm) and reduced ejection fraction (LVEF: 33%). The mildly dilated ascending aorta (45 mm) warrants monitoring but is not currently severe. Additionally, the reduced TAPSE (11 mm) and dilated IVC with poor collapsibility indicate right ventricular systolic dysfunction and elevated right atrial pressure, potentially secondary to advanced left-sided heart failure. Despite the significant left ventricular dilation and dysfunction, the left atrial volume index is normal (31 ml/m²), which is somewhat atypical for advanced heart failure and may reflect variability in remodeling or duration of disease. Pulmonary pressures appear mildly elevated, as suggested by TR Vmax (44 cm/s). |
| **Recommendations** | 1. Heart Failure Management:   Initiate or optimize guideline-directed medical therapy (GDMT) for heart failure with reduced ejection fraction (HFrEF), including: ACE inhibitors/ARBs or ARNI (e.g., sacubitril/valsartan), Beta-blockers (e.g., carvedilol, bisoprolol) titrated to target doses, Mineralocorticoid receptor antagonists (e.g., spironolactone), SGLT2 inhibitors (e.g., dapagliflozin) to improve outcomes in HFrEF   1. Diuretics for Symptom Relief:   Prescribe loop diuretics (e.g., furosemide) to manage volume overload and alleviate dyspnea. Monitor for electrolyte imbalances and renal function   1. Cardiac Resynchronization Therapy (CRT):   Evaluate for CRT if QRS duration is prolonged (>130 ms) on ECG, as this patient may benefit from improved cardiac efficiency   1. Monitoring and Imaging:   Regular echocardiographic follow-up every 6–12 months to assess LV size, ejection fraction, and right ventricular function. Serial imaging of the ascending aorta to monitor for further dilation   1. Advanced Heart Failure Therapies:   Consider referral to a heart failure specialist for evaluation of advanced therapies (e.g., ventricular assist devices or heart transplantation) if symptoms persist despite optimized medical therapy   1. Lifestyle and Risk Modification:   Advise on sodium and fluid restriction to mitigate congestion. Strongly recommend smoking cessation (if applicable) and stress management |
| **Conclusion** | This patient presents with dilated cardiomyopathy and right ventricular dysfunction, contributing to significant functional impairment (NYHA III). Immediate initiation of GDMT and symptomatic management is essential to improve outcomes. Close follow-up and advanced therapy evaluation are recommended, with consideration for CRT or transplant if medical management fails. |

| **Case** | **Case #5 (Mitral stenosis): male, 66 years old, no symptoms** |
| --- | --- |
| **Findings** | 1. **Left Ventricle (LV):**   **Interventricular Septal Thickness (IVSD):** 14 mm (mild hypertrophy)  **Left Ventricular End-Diastolic Dimension (LVEDD):** 56 mm (upper normal range)  **Left Ventricular Ejection Fraction (LVEF):** 45% (mildly reduced systolic function)   1. **Right Ventricle (RV):**   **Basal Diameter:** 35 mm (normal)  **TAPSE (Tricuspid Annular Plane Systolic Excursion):** 16 mm (normal RV systolic function)   1. **Valves:**   **Aortic Valve:** **mean Gradient:** 12 mmHg (suggests mild aortic stenosis)  **Mitral Valve:** **mean Gradient:** 10 mmHg (moderate mitral stenosis, possibly from rheumatic heart disease)  **TR Vmax (Tricuspid Regurgitation Max Velocity):** 34 m/s (mildly elevated, possible mild pulmonary hypertension)   1. Hemodynamics:   **Inferior Vena Cava (IVC):** 12 mm (normal size) |
| **Interpretation** | 1. **Mild Left Ventricular Dysfunction:**   The mildly reduced LVEF (45%) suggests some degree of left ventricular systolic dysfunction, which may be related to the long-term effects of **rheumatic heart disease** or other undiagnosed causes. The interventricular septal hypertrophy (IVSD of 14 mm) further suggests chronic pressure or volume overload   1. **Moderate Mitral Stenosis:**   The history of rheumatic fever and the mitral valve mean gradient of 10 mmHg are consistent with **rheumatic mitral valve stenosis**. Rheumatic heart disease can cause progressive valvular thickening and dysfunction over time   1. **Mild Aortic Stenosis:**   The aortic valve mean gradient of 12 mmHg indicates **mild aortic stenosis**, which could also be rheumatic in origin, as multi-valvular involvement is common in such cases   1. **Mild Pulmonary Hypertension:**   The tricuspid regurgitation velocity (TR Vmax of 34 m/s) suggests **mild pulmonary hypertension**, likely secondary to the mitral valve stenosis |
| **Recommendations** | 1. **Management of Valvular Disease:**   The patient should be evaluated for potential **medical or surgical management** of **mitral valve stenosis**. A **transesophageal echocardiogram (TEE)** or **mitral valve area assessment** may be useful for further quantifying the stenosis severity. As both the aortic and mitral valves are involved, a **multi-valvular assessment** should be undertaken to determine the timing and necessity of valve repair or replacement. Given the moderate severity of mitral stenosis, the patient may benefit from **mitral valve replacement** or **percutaneous balloon mitral valvuloplasty** depending on the severity of symptoms or progression   1. **Management of Left Ventricular Dysfunction:**   **Pharmacologic therapy** for heart failure, including **beta-blockers**, **ACE inhibitors**, or **angiotensin II receptor blockers (ARBs)**, may help to manage the reduced LV systolic function and prevent further deterioration   1. **Monitoring Pulmonary Hypertension:**   The mild pulmonary hypertension should be monitored over time. If symptoms such as dyspnea develop, further evaluation with **right heart catheterization** might be warranted   1. **Follow-up and Lifestyle Recommendations:**   Regular follow-up with echocardiographic assessments every **6 to 12 months** is recommended to monitor the progression of valvular disease and LV function. Although asymptomatic, the patient should be counseled on the symptoms of worsening valvular disease, such as increasing shortness of breath, fatigue, or palpitations, and encouraged to seek timely medical attention |
| **Conclusion** | This 66-year-old male, with a history of childhood rheumatic fever, demonstrates findings of **rheumatic heart disease** with **moderate mitral stenosis**, **mild aortic stenosis**, and **mildly reduced left ventricular systolic function**. Mild pulmonary hypertension is also present, likely secondary to mitral stenosis. Ongoing monitoring, medical management, and possible consideration of surgical or percutaneous intervention for mitral valve disease are recommended. |

| **Case** | **Case #6 (Cor pulmonale): female, 77 years old, dyspnoe NYHA II-III** |
| --- | --- |
| **Findings** | 1. **Left Ventricle (LV):**   **Interventricular Septal Thickness (IVSD):** 15 mm (mild hypertrophy)  **Left Ventricular End-Diastolic Dimension (LVEDD):** 44 mm (normal range)  **Left Ventricular Ejection Fraction (LVEF):** 50% (borderline systolic function)   1. **Right Ventricle (RV):**   **Basal Diameter:** 47 mm (mildly enlarged RV)  **TAPSE (Tricuspid Annular Plane Systolic Excursion):** 22 mm (normal RV systolic function)   1. **Valves:**   **Aortic Valve:** **mean Gradient:** 4 mmHg (normal)  **Mitral Valve:** **mean Gradient:** 3 mmHg (within normal limits)  **TR Vmax (Tricuspid Regurgitation Max Velocity):** 60 m/s (severe pulmonary hypertension)   1. Hemodynamics:   **Inferior Vena Cava (IVC):** 27 mm (dilated, suggestive of elevated right atrial pressure) |
| **Interpretation** | 1. **Borderline Left Ventricular Function:**   The LVEF of 50% indicates **borderline systolic function**, which may contribute to the patient’s dyspnea (NYHA II-III). The interventricular septal thickness (IVSD of 15 mm) is suggestive of **mild left ventricular hypertrophy**, possibly related to chronic pressure overload, possibly due to pulmonary hypertension from the underlying COPD   1. **Severe Pulmonary Hypertension:**   The tricuspid regurgitation velocity (TR Vmax of 60 m/s) is significantly elevated, indicating **severe pulmonary hypertension**. This finding is likely secondary to the patient’s **COPD**, as chronic lung disease is a common cause of elevated pulmonary pressures   1. **Right Ventricular Enlargement:**   The enlarged right ventricle (RV basal diameter of 47 mm) reflects the impact of chronic pressure overload due to pulmonary hypertension. The normal TAPSE of 22 mm suggests that **RV systolic function** is still preserved   1. **Dilated Inferior Vena Cava:**   The dilated IVC (27 mm) with limited respiratory collapse is consistent with **elevated right atrial pressure**, likely secondary to pulmonary hypertension |
| **Recommendations** | 1. **Management of Pulmonary Hypertension:**   Given the severe pulmonary hypertension, further investigation into the **pulmonary pressure dynamics** and evaluation of the underlying COPD is recommended. A **right heart catheterization** may help confirm the diagnosis and quantify the severity of pulmonary hypertension. **Pulmonary vasodilators** such as **phosphodiesterase-5 inhibitors** (e.g., sildenafil) or **endothelin receptor antagonists** (e.g., bosentan) could be considered if appropriate   1. **Optimizing COPD Management:**   The patient’s **COPD** is likely a contributing factor to the pulmonary hypertension and RV enlargement. Optimizing **COPD treatment** with bronchodilators, **inhaled corticosteroids**, and ensuring **smoking cessation** (if applicable) is crucial. **Pulmonary rehabilitation** should also be considered to improve overall lung function and symptoms   1. **Monitoring Right Ventricular Function:**   Regular monitoring of RV function is recommended, as right heart failure can develop over time in patients with chronic pulmonary hypertension. **Serial echocardiograms** every 6–12 months should be performed to track any deterioration   1. **Assessment of Left Ventricular Function:**   The **borderline LV systolic function** (LVEF of 50%) should be monitored. Optimizing **afterload reduction** with ACE inhibitors or ARBs, if not already prescribed, may be considered to prevent further LV dysfunction   1. **Follow-up and Lifestyle:**   Close follow-up with a **cardiologist** and **pulmonologist** is advised. Lifestyle changes, such as **low-sodium diet** and **fluid restriction**, may help in managing pulmonary hypertension symptoms |
| **Conclusion** | This 77-year-old female with a history of **COPD** and dyspnea (NYHA II-III) presents with **severe pulmonary hypertension** and **borderline LV systolic function**. The patient has significant **right ventricular enlargement** with preserved RV function. Management should focus on optimizing COPD therapy, addressing pulmonary hypertension, and closely monitoring for the development of right heart failure. |

| **Case** | **Case #7 (Hypertensive heart disease): male, 50 years old, no symptoms** |
| --- | --- |
| **Findings** | 1. **Left Ventricle (LV):**   **Interventricular Septal Thickness (IVSD):** 16 mm (mild hypertrophy)  **Left Ventricular End-Diastolic Dimension (LVEDD):** 54 mm (normal range)  **Left Ventricular Ejection Fraction (LVEF):** 60% (normal systolic function)   1. **Right Ventricle (RV):**   **Basal Diameter:** 39 mm (normal)  **TAPSE (Tricuspid Annular Plane Systolic Excursion):** 20 mm (normal RV systolic function)   1. **Valves:**   **Aortic Valve:** **mean Gradient:** 8 mmHg (normal)  **Mitral Valve:** **mean Gradient:** 3 mmHg (normal)  **Tricuspidal Valve: TR Vmax (Tricuspid Regurgitation Max Velocity):** 21 m/s (normal)   1. **Diastolic Function:**   **E/A Ratio:** 1.6 (normal to slightly elevated, indicative of mild diastolic dysfunction), **E/e’:** 7.6 (normal)   1. Hemodynamics:   **Inferior Vena Cava (IVC):** 16 mm (normal size) |
| **Interpretation** | 1. **Mild Left Ventricular Hypertrophy:**   The increased interventricular septal thickness (16 mm) suggests **mild left ventricular hypertrophy** (LVH). This could be related to the patient's history of smoking, possible undiagnosed hypertension, or chronic stress, both of which are risk factors for LVH.   1. **Normal Left Ventricular Systolic Function:**   Despite the LVH, the patient has **normal systolic function** with an LVEF of 60%.   1. **Normal Right Ventricular Function:**   The right ventricular size and function are within normal limits as indicated by the RV basal diameter and TAPSE measurements.   1. **Mild Diastolic Dysfunction:**   The E/A ratio of 1.6 with a normal E/e' of 7.6 suggests **mild diastolic dysfunction**, potentially an early sign of increased LV filling pressure, though this is compensated at this stage.   1. **No Significant Valve Pathology:**   The aortic and mitral valves show no significant abnormalities with normal gradients across both valves.   1. **Normal Pulmonary Pressures and IVC:**   The TR Vmax and IVC size indicate **normal pulmonary pressures** and **normal right atrial pressure**. |
| **Recommendations** | 1. **Monitor for Hypertension:**   Given the presence of **mild LV hypertrophy**, the patient should be monitored for **hypertension**. A **24-hour blood pressure monitor** may be considered to detect any stress-induced or masked hypertension that could contribute to LVH   1. **Lifestyle Modification:**   The patient's smoking habit and stressful job are key modifiable risk factors. **Smoking cessation** and **stress management** strategies should be strongly encouraged to reduce the risk of future cardiovascular disease. Referral to a **smoking cessation program** and stress reduction techniques (such as **exercise** or **mindfulness-based interventions**) would be beneficial   1. **Annual Echocardiography:**   Although there is no evidence of heart failure or significant valve disease, regular **echocardiographic follow-up** (every 1–2 years) is recommended to monitor for any progression of **LV hypertrophy** or diastolic dysfunction   1. **Screen for Metabolic Risk Factors:**   Screening for **dyslipidemia**, **diabetes**, and other cardiovascular risk factors is recommended. Given the smoking history and stressful lifestyle, a **cardiovascular risk assessment** could be valuable |
| **Conclusion** | This 50-year-old male smoker with no current symptoms has evidence of **mild left ventricular hypertrophy** and **mild diastolic dysfunction**, but otherwise normal cardiac function. The patient is at risk for future cardiovascular complications, particularly related to smoking and lifestyle stress. Close monitoring and lifestyle modification are key to preventing disease progression. |

| **Case** | **Case #8 (Amyloidosis): male, 86 years, proBNP 6000 ng/l, dyspnoe NYHA III, history of 3 x syncope, carpal tunnel syndrome** |
| --- | --- |
| **Findings** | 1. Left Ventricle (LV):   Interventricular Septum Thickness (IVSD): 16 mm (hypertrophied with a "bright" appearance suggestive of amyloidosis)  Left Ventricular End-Diastolic Dimension (LVEDD): 52 mm (normal range)  Left Ventricular End-Systolic Dimension (LVESD): 39 mm (normal range)  Left Ventricular Ejection Fraction (LVEF): 58% (normal)   1. Aorta: Ascending Aorta Diameter: 39 mm (upper limit of normal) 2. Left Atrial Volume Index (LAVI): 65 ml/m² (severely dilated) 3. Right Ventricle (RV):   Basal Diameter: 43 mm (mildly dilated)  TAPSE: 16 mm (borderline reduced, indicative of mildly reduced RV function)   1. Valves:   Aortic Valve: mean Gradient: 10 mmHg (suggestive of mild aortic stenosis)  Mitral Valve mean Gradient: 1 mmHg (normal)  Tricuspid Valve: TR Vmax: 49 m/s (suggestive of severe tricuspid regurgitation with high pulmonary pressures)   1. Diastolic Function:   E/A Ratio: 2.8 (suggestive of restrictive filling pattern), E/e’ Ratio: 19 (elevated, indicating increased LV filling pressures), Medial e': 9 cm/s, Lateral e': 12 cm/s   1. Hemodynamics:   Inferior Vena Cava: Diameter: 9 mm (small, potentially indicating low central venous pressure or volume depletion) |
| **Interpretation** | 1. Cardiac Amyloidosis:   The "bright" appearance of the myocardium, combined with interventricular septal hypertrophy, elevated proBNP, and diastolic dysfunction (restrictive filling pattern), is highly suggestive of cardiac amyloidosis   1. Heart Failure with Preserved Ejection Fraction (HFpEF):   The patient exhibits severe diastolic dysfunction (high E/e' ratio and severely dilated left atrium), consistent with HFpEF   1. Pulmonary Hypertension   The elevated TR Vmax suggests significant pulmonary hypertension, likely secondary to left heart disease   1. Aortic Stenosis   Mild aortic stenosis is present, although not hemodynamically significant at this stage   1. Right Ventricular Dysfunction:   Mild RV dilation and borderline TAPSE suggest early right ventricular involvement, possibly secondary to pulmonary hypertension or direct myocardial involvement (e.g., amyloidosis) |
| **Recommendations** | 1. Further Diagnostic Workup:   Cardiac MRI or Endomyocardial Biopsy: To confirm the diagnosis of cardiac amyloidosis. Serum/Urine Electrophoresis and Immunofixation: To identify light chains if AL amyloidosis is suspected. Genetic Testing: If transthyretin (ATTR) amyloidosis is suspected, especially in the context of carpal tunnel syndrome   1. Treatment:   Heart Failure Management: Initiate or optimize treatment for HFpEF, including diuretics to manage congestion, and potentially consider advanced therapies specific to amyloidosis. Pulmonary Hypertension: Monitor and manage secondary pulmonary hypertension; consider right heart catheterization for further evaluation. Aortic Stenosis: Continue to monitor, given the mild gradient. Surgical or percutaneous intervention may be required if symptoms progress   1. Follow-up:   Close Monitoring: Regular follow-up for worsening heart failure symptoms, arrhythmias, or progression of valvular disease. Referral to a specialist: Given the complexity and suspicion of amyloidosis, referral to a cardiologist specializing in amyloidosis is recommended |
| **Conclusion** | The patient likely has cardiac amyloidosis with heart failure with preserved ejection fraction (HFpEF) and secondary pulmonary hypertension. Urgent further evaluation and initiation of amyloidosis-specific therapies are advised. |

| **Case** | **Case #9 (Illogical): female, 40 years old, no symptoms** |
| --- | --- |
| **Findings** | 1. **Left Ventricle (LV):**   Left Ventricular End-Diastolic Dimension (**LVEDD):** 10 mm (This appears unusually low, likely a measurement error or transcription mistake)  Left Ventricular End-Systolic Dimension (**LVESD):** 39 mm  Left Ventricular Ejection Fraction (**LVEF):** 58%   1. **Aorta: Ascending Aorta Diameter:** 60 cm (This value is inconsistent and seems excessively large for any patient. A value in millimeters or centimeters within the normal range for the aorta is expected.) 2. **Left Atrium Volume Index (LAVI):** 25 ml/m² 3. **Right Ventricle (RV):**   **RV Basal Diameter:** 37 mm  **TAPSE:** 23 mm   1. Valves:   **Aortic Valve: AV Mean Pressure Gradient:** 10 mmHg  **Mitral Valve: MV Mean Pressure Gradient:** 1 mmHg  **Tricuspid Valve: Regurgitation: TR Vmax:** 2.4 m/s   1. **Diastolic Function:**   **E/A Ratio:** 1.1, **E/e’ Ratio:** 7, **Medial e’:** 9 cm/s, **Lateral e’:** 12 cm/s   1. Hemodynamics:   **Inferior Vena Cava (IVC): Diameter:** 9 mm |
| **Interpretation** | 1. **Left Ventricle (LV):**   The LVEDD measurement of 10 mm is highly inconsistent with normal physiological values and likely reflects a measurement error. For a 36-year-old female, a normal LVEDD is expected to be around 37-50 mm. The LVESD and LVEF values suggest normal systolic function, assuming the LVEDD is incorrect   1. **Aorta**   The measurement of 60 cm for the ascending aorta is also inconsistent and not physiologically plausible. This value should be reassessed as it is likely a significant error in measurement or transcription. Typically, a normal ascending aorta measures around 20-37 mm   1. **Left Atrium:**   The left atrium is within normal size limits, with no evidence of left atrial enlargement   1. **Right Ventricle (RV):**   The right ventricle is of normal size, and the TAPSE value indicates preserved systolic function   1. **Aortic Valve:**   The aortic valve gradient is within normal limits, with no significant aortic stenosis   1. **Mitral Valve:**   The mitral valve pressure gradient is normal, indicating no significant mitral stenosis   1. **Tricuspid Regurgitation:**   The tricuspid regurgitation velocity is within normal limits, suggesting no significant pulmonary hypertension.   1. **Diastolic Function:**   The diastolic function appears normal or at most mildly impaired, as indicated by the E/A and E/e’ ratios.   1. **Inferior Vena Cava (IVC):**   The IVC diameter is slightly on the lower side, which could indicate a lower intravascular volume, but it is not concerning given the overall context of the other measurements. |
| **Recommendations** | 1. **Re-evaluation:** It is essential to repeat the echocardiogram to correct the obvious measurement errors, particularly for the LVEDD and ascending aorta. 2. **Monitoring:** Routine follow-up with echocardiography may be necessary, particularly after correcting the erroneous measurements, to ensure accurate assessment of cardiac function. 3. **Lifestyle Advice:** Continue with heart-healthy lifestyle practices, including regular exercise, a balanced diet, and smoking cessation (if applicable). 4. **No Immediate Treatment Required:** Assuming the corrected measurements fall within normal ranges, no specific cardiac treatment is needed at this time. |
| **Conclusion** | This patient likely has a normal cardiovascular profile, but re-assessment is necessary due to the measurement inconsistencies. |

| **Case** | **Case #10 (Illogical): male, 40 years old, no symptoms** |
| --- | --- |
| **Findings** | 1. Left Ventricle (LV):   IVSd (Interventricular Septum Diastole): 10 mm (normal)  LVEDD (Left Ventricular End-Diastolic Dimension): 57 mm (mildly dilated, upper limit for males ~56 mm)  LVEF (Left Ventricular Ejection Fraction): 47% (reduced, indicative of mildly impaired systolic function)   1. Left Atrial Volume Index (LAVI): 28 ml/m² (normal, <34 ml/m²) 2. Aorta: Ascending Aorta Diameter: 34 mm (normal, <37 mm for males) 3. Right Ventricle (RV):   RV Basal Diameter: 38 mm (normal, <42 mm)  TAPSE (Tricuspid Annular Plane Systolic Excursion): 20 mm (normal, >17 mm)   1. Valves:   Aortic Valve: mean gradient: 4 mmHg (normal), effective Orifice Area (EOA): 3.4 cm² (normal, >2 cm²)  Mitral Valve: mean gradient: 1 mmHg (normal, <5 mmHg)  Tricuspid Valve: TR Gradient: 15 mmHg (normal). No significant regurgitation or stenosis noted   1. Diastolic Function:   E/A Ratio: 1.4 (normal, 0.8–2.0), E/e’: 8 (normal, <14), Medial e’: 7 cm/s; Lateral e’: 8 cm/s (within normal)   1. Hemodynamics:   Inferior Vena Cava (IVC): Diameter: 16 mm (normal, 15–21 mm with normal collapsibility)  SVI (Stroke Volume Index): 60 ml/m² (elevated, normal 35–50 ml/m²) |
| **Interpretation** | 1. Mild Left Ventricular Dilation (LVEDD at upper limit of normal) with mildly reduced systolic function (LVEF 47%). 2. Normal diastolic function, atrial size, right ventricular size/function, and valve hemodynamics. 3. Elevated stroke volume index may suggest a high-output state, though it needs clinical correlation (e.g., hyperdynamic circulation or anemia). |
| **Recommendations** | 1. Treatment/Follow-up:   Address possible causes of reduced ejection fraction, such as: Blood pressure control (if hypertensive). Lifestyle changes: Regular aerobic exercise and a heart-healthy diet. Consider beta-blockers and/or ACE inhibitors if clinically indicated, even in asymptomatic LV dysfunction. Monitor for potential symptoms of heart failure or progression (e.g., dyspnea, fatigue, exercise intolerance)   1. Monitoring:   Repeat echocardiography in 3–6 months to monitor left ventricular size and function. Consider assessment of myocardial viability or coronary artery disease if risk factors are present (e.g., ischemic cardiomyopathy as a potential cause)   1. Additional Workup:   Evaluate for potential causes of reduced EF (e.g., prior silent myocardial infarction, viral myocarditis, or cardiomyopathy). Laboratory tests: Complete blood count, NT-proBNP, and thyroid function tests |
| **Conclusion** | This 40-year-old asymptomatic male has evidence of mildly reduced LV systolic function and borderline LV dilation without overt signs of heart failure. Follow-up imaging and clinical evaluation are recommended to determine progression or resolution and identify underlying causes. |

| **Case** | **Case #11 (low-flow-low-gradient aortic stenosis and atrial fibrillation): female, 78 years old, Dyspnoe NYHA II-III, fatigue** |
| --- | --- |
| **Findings** | 1. Left Ventricle (LV):   IVSd (Interventricular Septum Diastole): 15 mm (hypertrophied, >12 mm)  LVEDD (Left Ventricular End-Diastolic Dimension): 49 mm (normal, 42–54 mm for females)  LVEF (Left Ventricular Ejection Fraction): 30% (severely reduced, indicating systolic dysfunction)   1. Aorta: Ascending Aorta Diameter: 36 mm (normal, <37 mm for females) 2. Left Atrial Volume Index (LAVI): 40 ml/m² (enlarged, normal <34 ml/m²) 3. Right Ventricle (RV):   RV Basal Diameter: 32 mm (normal, <42 mm)  TAPSE (Tricuspid Annular Plane Systolic Excursion): 13 mm (reduced, <17 mm indicates impaired RV systolic function)   1. Valves   Aortic Valve: Mean Gradient: 23 mmHg (moderately elevated, indicating stenosis), Effective Orifice Area (EOA): 0.8 cm² (severe aortic stenosis, <1.0 cm²)  Mitral Valve: Mean gradient: 1 mmHg (normal, <5 mmHg)  Tricuspid Valve: TR Gradient: 30 mmHg (normal to mild pulmonary hypertension, cutoff >35 mmHg).   1. Diastolic Function:   E/e’: 15 (elevated, suggests elevated left ventricular filling pressures), Medial e’: 5 cm/s, Lateral e’: 7 cm/s (reduced, indicating impaired relaxation)   1. Hemodynamics:   Inferior Vena Cava (IVC): Diameter: 18 mm (normal)  SVI (Stroke Volume Index): 32 ml/m² (reduced, normal 35–50 ml/m²) |
| **Interpretation** | This 78-year-old female with dyspnea (NYHA II-III) and fatigue has findings consistent with severe aortic stenosis, severely reduced left ventricular ejection fraction (HFrEF), LV hypertrophy, and elevated left atrial pressures. Additionally, there is evidence of mild right ventricular dysfunction (reduced TAPSE) and a reduced stroke volume. The combination of severe aortic stenosis and reduced LV function likely explains the patient’s symptoms and elevated filling pressures. These findings are consistent with heart failure with reduced ejection fraction (HFrEF) and severe valvular heart disease. |
| **Recommendations** | 1. Urgent Management of Aortic Stenosis:   Refer for heart team evaluation for consideration of aortic valve replacement: Surgical aortic valve replacement (SAVR) or transcatheter aortic valve replacement (TAVR), depending on patient comorbidities and surgical risk   1. Heart Failure Management:   Optimize heart failure therapy: ACE inhibitors/ARBs or ARNi: If not contraindicated, to reduce afterload and improve LV function. Beta-blockers: Start low and titrate carefully if tolerated. Mineralocorticoid receptor antagonists (MRA): If potassium levels allow.Diuretics: For symptom relief of congestion   1. Monitoring:   Regular follow-up with repeat echocardiography after intervention to assess LV recovery. Monitor for worsening symptoms (e.g., progression to NYHA IV) or complications such as arrhythmias   1. Lifestyle Modifications:   Sodium restriction and fluid balance monitoring. Regular physical activity as tolerated |
| **Conclusion** | The patient presents with severe symptomatic aortic stenosis, systolic heart failure (HFrEF), and secondary LV hypertrophy. Urgent evaluation for valve replacement is critical to improve symptoms and prognosis. |

| **Case** | **Case #12: male, 60 years old, no symptoms** |
| --- | --- |
| **Findings** | 1. Left Ventricle (LV):   IVSd (Interventricular Septum): 14 mm (mild hypertrophy, normal ≤12 mm)  LVEDD (Left Ventricular End-Diastolic Dimension): 56 mm (upper limit of normal, <56 mm for males)  LVEF (Left Ventricular Ejection Fraction): 55% (normal, 52–72%)   1. Aorta: Ascending Aorta Diameter: 34 mm (normal, <37 mm for males) 2. Left Atrial Volume Index (LAVI): 32 ml/m² (normal, <34 ml/m²) 3. Right Ventricle (RV):   RV Basal Diameter: 39 mm (normal, <42 mm)  TAPSE (Tricuspid Annular Plane Systolic Excursion): 14 mm (reduced, <17 mm suggests impaired RV systolic function)   1. Valves:   Aortic Valve: Mean gradient: 8 mmHg (normal, <10 mmHg). Effective Orifice Area (EOA): 3.1 cm² (normal, >2.0 cm²)  Mitral Valve: Mean gradient: 2 mmHg (normal, <5 mmHg)  Tricuspid Valve: TR Gradient: 15 mmHg (normal, <35 mmHg)   1. Diastolic Function:   E/A Ratio: 0.9 (impaired relaxation, E/A <1 suggests Grade I diastolic dysfunction). E/e’: 12 (normal, <14, but on the higher side indicating borderline filling pressures). Medial e’: 5 cm/s and Lateral e’: 8 cm/s (reduced medial velocity, consistent with impaired relaxation)   1. Hemodynamics:   Inferior Vena Cava (IVC): Diameter: 14 mm (normal size, collapsibility not mentioned)  Stroke Volume Index (SVI): 50 ml/m² (upper limit of normal, 35–50 ml/m²) |
| **Interpretation** | - 1. Mild left ventricular hypertrophy (likely related to hypertension or diabetic cardiomyopathy).   2. Preserved ejection fraction with impaired diastolic relaxation (Grade I diastolic dysfunction) and borderline filling pressures (E/e’ = 12)   3. Normal left atrial size and no significant valvular dysfunction   4. Mildly reduced right ventricular systolic function (TAPSE 14 mm)   5. Stroke volume index at the upper limit of normal, which may reflect hyperdynamic circulation (clinical correlation needed). |
| **Recommendations** | 1. Treatment/Management:   Optimize cardiovascular risk factor management: Strict blood pressure control (target <130/80 mmHg). Optimize diabetes control (HbA1c target individualized, ideally <7.0%). Smoking cessation counseling and support. Consider starting or adjusting medications: ACE inhibitors/ARBs: For blood pressure and potential myocardial remodeling benefits. Statin therapy: If not already prescribed, for secondary prevention given the smoking and diabetes history   1. Lifestyle Modifications:   Encourage regular moderate aerobic exercise to improve diastolic function. Heart-healthy diet (low in saturated fats and salt)   1. Monitoring/Follow-Up:   Repeat echocardiography in 6–12 months to monitor LV function, diastolic parameters, and RV systolic function (TAPSE). Monitor for symptoms of heart failure or worsening exercise tolerance. Periodic evaluation of cardiovascular risk factors (e.g., lipids, glycemic control)   1. Additional Considerations:   Further assess for subclinical coronary artery disease (e.g., coronary calcium scoring or functional testing), given the diabetes and smoking history |
| **Conclusion** | This patient demonstrates mild cardiac abnormalities, including mild LV hypertrophy, impaired diastolic relaxation, and reduced TAPSE. These findings are likely related to longstanding cardiovascular risk factors (diabetes and smoking). Close follow-up and aggressive risk factor management are essential to prevent disease progression. Currently, no urgent interventions are required. |

| **Case** | **Case #13: female, 72 years old, fatigue** |
| --- | --- |
| **Findings** | 1. **Left Ventricle (LV):**   **IVSd (Interventricular Septum):** 12 mm (normal, <12 mm for females; borderline for mild hypertrophy)  **LVEDD (Left Ventricular End-Diastolic Dimension):** 49 mm (normal, 42–54 mm for females)  **LVEF (Left Ventricular Ejection Fraction):** 55% (normal, 52–72%)   1. **Aorta: Ascending Aorta Diameter:** 37 mm (upper normal limit, <37 mm for females) 2. **Left Atrial Volume Index (LAVI):** 30 ml/m² (normal, <34 ml/m²) 3. **Right Ventricle (RV):**   **RV Basal Diameter:** 39 mm (normal, <42 mm)  **TAPSE (Tricuspid Annular Plane Systolic Excursion):** 18 mm (normal, ≥17 mm)   1. **Valves:**   **Aortic Valve:** Mean gradient: 5 mmHg (normal, <10 mmHg). Effective Orifice Area (EOA): 3.0 cm² (normal, >2.0 cm²)  **Mitral Valve:** Mean gradient: 2 mmHg (normal, <5 mmHg)  **Tricuspid Valve TR Gradient:** 28 mmHg (normal to mild pulmonary pressures, <35 mmHg)   1. **Diastolic Function:**   **E/e’:** 17 (elevated, suggests increased LV filling pressures). **Medial e’:** 5 cm/s (reduced, indicating impaired myocardial relaxation). **Lateral e’:** 14 cm/s (normal)   1. **Hemodynamics:**   **Inferior Vena Cava (IVC):** Diameter: 25 mm (dilated, >21 mm; suggests elevated right atrial pressure if collapsibility is reduced)  **Stroke Volume Index (SVI):** 39 ml/m² (normal, 35–50 ml/m²) |
| **Interpretation** | 1. **Normal LV systolic function** (LVEF = 55%) with no evidence of LV dilation or significant hypertrophy. 2. **Impaired diastolic relaxation** (reduced medial e’, elevated E/e’ = 17), consistent with **Grade II diastolic dysfunction** and **elevated left ventricular filling pressures**. 3. **Mildly elevated right atrial pressures**, suggested by a dilated IVC (25 mm) and elevated E/e’. 4. **Normal valve function** with no significant stenosis or regurgitation. 5. **Mildly elevated TR gradient** but no evidence of pulmonary hypertension. |
| **Recommendations** | 1. **Treatment:**   **Diastolic Dysfunction Management:** Ensure optimal blood pressure control (target <130/80 mmHg), preferably with **ACE inhibitors/ARBs** or calcium channel blockers. Consider diuretics (e.g., furosemide) if there are signs of fluid overload or congestion. Address fatigue by improving cardiovascular fitness through supervised exercise tailored to her capacity. Optimize treatment of potential contributing factors (e.g., ischemia, anemia, thyroid dysfunction)   1. **Lifestyle Modifications:**   Sodium-restricted diet to reduce volume overload. Encourage weight management and physical activity to improve symptoms and diastolic function   1. **Monitoring/Follow-Up:**   Periodic echocardiographic follow-up to monitor diastolic parameters and right atrial pressures. Clinical follow-up for symptom progression or development of heart failure   1. **Further Investigations:**   Assess for comorbidities contributing to diastolic dysfunction: Coronary artery disease (e.g., stress testing or coronary CTA). Thyroid function, anemia, or other systemic causes of fatigue |
| **Conclusion** | The findings are consistent with **Grade II diastolic dysfunction** leading to **elevated filling pressures**, likely contributing to the patient's fatigue. Management should focus on optimizing blood pressure, volume status, and addressing underlying comorbidities. Regular follow-up is recommended to monitor for progression or the development of symptoms suggestive of heart failure. |

**Table S1: LLM-generated report of fictional cases (1-13, Group 1)**

| **Case** | **Case #14 (healthy): male, 18 years old, no symptoms** |
| --- | --- |
| **Findings** | 1. Left Ventricle (LV):   IVSd (Interventricular Septum): 10 mm (normal, <11 mm for males)  LVEDD (Left Ventricular End-Diastolic Dimension): 48 mm (normal, 42–58 mm for males)  LVEF (Left Ventricular Ejection Fraction): 59% (normal, 52–72%)   1. Left Atrial Volume Index (LAVI): Not provided 2. Right Ventricle (RV):   RV Basal Diameter: 27 mm (normal, <42 mm)  TAPSE (Tricuspid Annular Plane Systolic Excursion): 18 mm (normal, ≥17 mm)   1. Valves:   Aortic Valve: Mean gradient: 4 mmHg (normal, <10 mmHg)  Mitral Valve: Mean gradient: 2 mmHg (normal, <5 mmHg)  Tricuspid Valve: TR Gradient: Not provided   1. Diastolic Function:   E/A Ratio: 2.15 (elevated, suggestive of increased early diastolic filling; can be normal in young adults), E/e’: 6.7 (normal, <10; suggests normal left ventricular filling pressures), Medial e’: 11.2 cm/s (normal, >7 cm/s), Lateral e’: 15.1 cm/s (normal, >10 cm/s)   1. Hemodynamics:   Inferior Vena Cava (IVC)/Stroke Volume Index (SVI): Not provided |
| **Interpretation** | 1. Normal left ventricular size and systolic function (LVEF = 59%) 2. No evidence of significant hypertrophy, dilation, or dysfunction 3. Normal diastolic function with a slightly elevated E/A ratio, which is typical in young adults due to high early diastolic filling velocities 4. Normal right ventricular size and systolic function (TAPSE = 18 mm) 5. No significant valvular abnormalities |
| **Recommendations** | 1. No Treatment Necessary:   No signs of heart disease; no interventions are required.   1. Monitoring/Follow-Up:   Routine follow-up is not required unless symptoms develop.   1. Lifestyle Recommendations:   Maintain a healthy lifestyle, including regular physical activity, a balanced diet, and avoiding smoking or excessive alcohol use. |
| **Conclusion** | The echocardiogram is normal. The patient shows no evidence of structural or functional heart disease. No treatment or monitoring is required unless symptoms arise. |

| **Case** | **Case #15 (Hypertensive heart disease): male, 80 years old, headache** |
| --- | --- |
| **Findings** | 1. **Left Ventricle (LV):**   **IVSd (Interventricular Septum):** 14 mm (mildly increased; normal is ≤12 mm, suggesting mild LV hypertrophy)  **LVEDD (Left Ventricular End-Diastolic Dimension):** 50 mm (normal range for males: ~42–58 mm)  **LVEF (Left Ventricular Ejection Fraction):** 60% (normal, 52–72%)   1. **Aorta: Ascending Aorta:** 34 mm (within normal limits, <37 mm for males) 2. **Right Ventricle (RV):**   **RV Basal Diameter:** 37 mm (normal, <42 mm)  **TAPSE (Tricuspid Annular Plane Systolic Excursion):** 18 mm (normal, ≥17 mm, indicating normal RV systolic function)   1. **Valves:**   **Aortic Valve:** Mean gradient: 3 mmHg (normal, <10 mmHg)  **Mitral Valve:** Mean gradient: 1 mmHg (normal, <5 mmHg)  Tricuspid Valve: **TR Gradient:** 22 mmHg (suggests normal right ventricular systolic pressure, generally <35 mmHg is considered normal or borderline).   1. **Diastolic Function:**   **E/A:** 1.47 (normal range roughly 1.0–2.0; consistent with normal or slightly enhanced early filling), **Medial e':** 10.5 cm/s and **Lateral e':** 17.2 cm/s (both within normal or high-normal limits, indicative of good diastolic relaxation at least at the lateral annulus), **E/e'**: Not provided (cannot directly assess filling pressures from this exam alone).   1. **Other Parameters: LAVI, SVI, EOA, IVC**: Not provided |
| **Interpretation** | 1. **Mild Left Ventricular Hypertrophy** is suggested by an interventricular septal thickness of 14 mm. This may be age-related or could reflect a history of hypertension (common in an 80-year-old), though blood pressure history is not provided. 2. **Normal LV Size and Systolic Function** (LVEF 60%) 3. **No Significant Valvular Disease**, as indicated by low mean gradients across the aortic and mitral valves 4. **Normal or Mildly Enhanced Diastolic Filling** pattern suggested by an E/A of 1.47 and normal-to-high e' velocities, although **E/e'** is not provided to confirm filling pressures 5. **Normal Right Ventricular Size and Function**, with TAPSE of 18 mm 6. **Normal Pulmonary Pressure** based on TR gradient of 22 mmHg |
| **Recommendations** | 1. **Blood Pressure and Cardiovascular Risk Management:**   Evaluate and manage possible hypertension (a common cause of LV hypertrophy), especially in an 80-year-old patient presenting with headaches. Ensure regular follow-up of blood pressure and cardiovascular status. Consider standard cardiovascular risk factor assessment (lipids, diabetes screening if not done, etc.)   1. **Lifestyle Measures:**   Encourage regular physical activity (as tolerated), a low-salt diet (if hypertensive), and smoking cessation if applicable. Promote heart-healthy nutrition and weight control   1. **Follow-Up:**   If hypertension is suspected or confirmed, echocardiographic re-evaluation in 6–12 months may be considered to assess any progression or regression of LV hypertrophy. If headaches are persistent or severe, consider alternative etiologies and neurologic evaluation   1. **Additional Echocardiographic Details (if clinically needed):**   **Left Atrial Volume Index (LAVI)** and **E/e'** to better characterize diastolic function. **Inferior Vena Cava (IVC) size and collapsibility** to assess volume status and right atrial pressure. **Stroke Volume Index (SVI)** for a more complete hemodynamic assessment |
| **Conclusion** | This 80-year-old male’s echocardiogram shows **mild LV hypertrophy** with **normal systolic function** and **no significant valvular abnormalities**. Diastolic function appears relatively preserved but incomplete due to missing E/e’ data. The primary clinical concern for headache may be related to hypertension or other non-cardiac factors. Regular blood pressure monitoring and follow-up are advised. |

| **Case** | **Case #16 (Moderate aortic valve stenosis): male, 87 years old, headache** |
| --- | --- |
| **Findings** | 1. **Left Ventricle (LV):**   **IVSd (Interventricular Septum):** 15 mm (mildly increased; normal upper limit ~12 mm, suggesting mild LV hypertrophy)  **LVEDD (Left Ventricular End-Diastolic Diameter):** 49 mm (normal range for an adult male)  **LVEF (Left Ventricular Ejection Fraction):** 55% (normal systolic function)   1. **Left Atrium Volume Index (LAVI):** 25 ml/m² (normal, <34 ml/m²) 2. **Right Ventricle (RV):**   **RV Basal Diameter:** 37 mm (normal, <42 mm)  **TAPSE (Tricuspid Annular Plane Systolic Excursion):** 22 mm (normal, ≥17 mm, indicating preserved RV systolic function)   1. Valves:   **Aortic Valve: Mean Gradient:** 21 mmHg (moderate gradient, suggestive of moderate aortic stenosis), **Effective Orifice Area (EOA):** 1.22 cm² (in the moderate stenosis range; typically 1.0–1.5 cm²)  **Mitral Valve (MV), Mean Gradient:** 2 mmHg (normal, <5 mmHg)  Tricuspid Valve: **TR Gradient:** 35 mmHg (upper-normal or mildly elevated, borderline for pulmonary hypertension)   1. **Diastolic Function:**   **E/A:** 0.80 (indicative of impaired relaxation; often seen in older adults or early diastolic dysfunction), **E/e’:** 13.5 (borderline elevated; normal <14, suggesting mildly increased LV filling pressure), **Medial e’:** 5.9 cm/s, **Lateral e’:** 6.1 cm/s (both slightly reduced, consistent with diastolic dysfunction). |
| **Interpretation** | 1. **Moderate Aortic Stenosis**: The mean gradient of 21 mmHg and EOA of 1.22 cm² place this patient in the moderate stenosis range. 2. **Mild Left Ventricular Hypertrophy**: IVSd of 15 mm is consistent with mild LVH, which may be related to age, hypertension, or the valvular stenosis. 3. **Preserved Systolic Function**: LVEF of 55% is within normal limits, indicating no overt systolic dysfunction. 4. **Diastolic Dysfunction**: E/A ratio <1 and borderline-elevated E/e’ suggest mild impaired relaxation and mildly raised filling pressures, often seen in older patients. 5. **Right Ventricular Function**: TAPSE is normal, signifying intact RV systolic function, though the TR gradient suggests borderline/mildly elevated pulmonary pressures. |
| **Recommendations** | 1. **Clinical Follow-Up**   Continue routine cardiovascular assessments, including blood pressure control and monitoring for the development of symptoms (e.g., exertional dyspnea, fatigue, chest pain, or syncope). If the patient develops symptoms suggestive of aortic stenosis progression, expedite re-evaluation.   1. **Repeat Echocardiogram**   Consider a follow-up echo in **6–12 months** or sooner if new symptoms arise to track progression of aortic stenosis and LV function.   1. **Risk Factor Management**   Address and optimize potential contributing factors such as hypertension and/or other cardiovascular risks (e.g., lipids, diabetes, smoking cessation if applicable). Encourage a healthy lifestyle: balanced diet, regular low- to moderate-intensity exercise appropriate for age and fitness level.   1. **Additional Testing**   If there is any suspicion of worsening stenosis or cardiac symptoms, further testing (e.g., stress echocardiography, cardiac CT) may be considered to clarify severity and management. |
| **Conclusion** | The patient demonstrates **moderate aortic stenosis** with preserved LV systolic function, mild LV hypertrophy, and mild diastolic dysfunction. He is currently asymptomatic. Ongoing surveillance is recommended to monitor for progression of stenosis or the onset of symptoms. |

| **Case** | **Case #17 (severe aortic valve stenosis): female, 78 years old, 2x syncope** |
| --- | --- |
| **Findings** | 1. **Left Ventricle (LV)**   **IVSd (Interventricular Septum):** 13 mm (mild concentric hypertrophy, normal upper limit ~11–12 mm for females  **LVEDD (End-Diastolic Diameter):** 41 mm (within normal limits, slightly on the lower side of normal range)  **LVEF (Left Ventricular Ejection Fraction):** 59% (normal systolic function)   1. **Aorta: Ascending Aorta Diameter:** 32 mm (normal for a female) 2. **Right Ventricle (RV):**   **RV Basal Diameter:** 30 mm (normal, <42 mm)  **TAPSE (Tricuspid Annular Plane Systolic Excursion):** 17 mm (lower limit of normal, indicative of borderline RV systolic function)   1. Valves:   **Aortic Valve (AV): Mean Gradient:** 42 mmHg (indicative of severe obstruction; severe aortic stenosis generally >40 mmHg), **Effective Orifice Area (EOA):** 0.95 cm² (severe aortic stenosis typically ≤1.0 cm²)  **Mitral Valve (MV): Mean Gradient:** 1 mmHg (normal, <5 mmHg)  **Tricuspid Valve: TR Gradient:** 28 mmHg (normal to mildly elevated; typically <30 mmHg considered normal or borderline)   1. **Diastolic Function:**   **E/A Ratio:** 0.50 (suggestive of impaired LV relaxation / Grade I diastolic dysfunction), **E/e’:** 7.9 (normal to borderline, indicating relatively normal LV filling pressures), **Medial e’:** 4.1 cm/s (low), **Lateral e’:** 7.8 cm/s (borderline-low) – consistent with impaired relaxation   1. **Inferior Vena Cava (IVC): Diameter:** 12 mm (within normal range, though precise collapsibility not reported). |
| **Interpretation** | 1. **Severe Aortic Stenosis**: The **mean gradient of 42 mmHg** and **EOA of 0.95 cm²** place this patient firmly in the severe aortic stenosis category 2. **Normal LV Systolic Function**: LVEF is 59% 3. **Mild Concentric LV Hypertrophy**: IVSd of 13 mm, common in long-standing valvular stenosis or hypertension (blood pressure history not provided) 4. **Grade I Diastolic Dysfunction**: Low E/A (0.50) indicates impaired relaxation; however, E/e’ is not significantly elevated, suggesting relatively normal filling pressures at rest 5. **Syncope**: The patient has had two episodes of syncope—a classic symptom that can be associated with severe aortic stenosis |
| **Recommendations** | 1. **Further Evaluation & Management of Severe Aortic Stenosis**   Given the **severe stenosis** (mean gradient >40 mmHg, EOA <1.0 cm²) and **symptoms (syncope)**, this patient meets criteria for **interventional treatment**. Refer to a **heart team** (cardiology and cardiothoracic surgery) for evaluation of **aortic valve replacement**. Options include **SAVR (Surgical Aortic Valve Replacement)** or **TAVR (Transcatheter Aortic Valve Replacement)**, depending on surgical risk and comorbidities.   1. **Medical Management Until Intervention**   Monitor for signs of heart failure (dyspnea, fatigue, exercise intolerance). Control blood pressure if hypertensive, aiming for cautious management to maintain adequate coronary perfusion. Avoid excessive vasodilators that could reduce preload/afterload significantly and exacerbate symptoms.   1. **Follow-Up**   Repeat echocardiography if there is any clinical change or post-intervention to assess valve function and LV remodeling. Continue regular cardiology evaluations, especially in the presence of syncope or other symptomatic episodes.   1. **Lifestyle & Risk Factor Modification**   Optimize cardiovascular risk factors (e.g., lipids, glucose) and encourage appropriate physical activity (low- to moderate-intensity based on clinical tolerance). Maintain normal sinus rhythm if arrhythmias are present (not indicated here but relevant in the broader context) |
| **Conclusion** | This 76-year-old female presents with **severe symptomatic aortic stenosis** (two syncopal episodes) and **preserved LV systolic function**. Mild LV hypertrophy and Grade I diastolic dysfunction are likely consequences of longstanding pressure overload. Definitive management with **aortic valve replacement** is strongly indicated given the severity of stenosis and the presence of syncope. |

| **Case** | **Case #18 (amyloidosis): male, 82 years old, dyspnoe NYHA II** |
| --- | --- |
| **Findings** | 1. **Left Ventricle (LV):**   **IVSd (Interventricular Septum):** 20 mm, significantly thickened (normal upper limit ~11–12 mm for females, ~12 mm for males). This suggests **marked LV hypertrophy** or possible **infiltrative process**  **LVEDD (End-Diastolic Diameter):** 41 mm, on the lower side of normal, indicating a **small LV cavity**  **LVEF (Left Ventricular Ejection Fraction):** 42%, mildly to moderately **reduced systolic function** (normal: ~52–72%)   1. **Left Atrium Volume Index (LAVI):** 29 ml/m², near the upper limit of normal (<34 ml/m²) 2. **Right Ventricle (RV):**   **RV Basal Diameter:** 43 mm, mildly enlarged (normal <42 mm)  **TAPSE (Tricuspid Annular Plane Systolic Excursion):** 15 mm, **reduced** (<17 mm indicates impaired RV systolic function)   1. **Valves:**   **Aortic Valve:** Mean gradient: 5 mmHg (normal range, suggesting no significant stenosis), EOA (Effective Orifice Area): 1.87 cm² (normal).  **Mitral Valve**: Mean gradient: 2 mmHg (normal)  Tricuspid Valve: **TR Gradient:** 21 mmHg Normal to mildly elevated (typically <30 mmHg is normal-borderline)   1. **Diastolic Function:**   **E/e′:** 20.0 (elevated, indicating **increased LV filling pressures**), **Medial e′:** 4.9 cm/s and **Lateral e′:** 6.0 cm/s (both low, consistent with **impaired myocardial relaxation**), **E/A**: Not provided.   1. **IVC Diameter**: 16 mm, within normal range (~15–21 mm), but collapsibility not specified |
| **Interpretation** | **Marked LV Wall Thickening** (IVSd 20 mm) with a **small LV cavity** (LVEDD 41 mm) and **mildly reduced LVEF (42%)** raises suspicion for:   - **Infiltrative Cardiomyopathy** (e.g., **Cardiac Amyloidosis**), especially given the history of **carpal tunnel syndrome** (a known association with transthyretin amyloidosis). - **Hypertrophic Cardiomyopathy** could also present with thick septum, but an infiltrative process is more likely in an older patient with associated carpal tunnel syndrome, borderline EF, and diastolic dysfunction.   **Diastolic Dysfunction** is evident from elevated filling pressures (E/e′=20) and low e′ velocities  **Right Ventricular Involvement** is suggested by mild dilation (RV basal 43 mm) and reduced TAPSE (15 mm)  **Valvular Function** is essentially normal, with no significant stenosis or regurgitation based on mean gradients  The patient’s symptom of **dyspnea (NYHA II)** can be explained by a combination of **LV diastolic dysfunction** and **mildly reduced systolic function** |
| **Recommendations** | 1. **Further Diagnostic Evaluation:**   Strongly consider workup for **Cardiac Amyloidosis**, given the classic triad of: Thickened ventricles, Diastolic dysfunction, Carpal tunnel syndrome **Advanced cardiac imaging** (e.g., cardiac MRI) or **99mTc-PYP scan** (for transthyretin amyloid) can help confirm or exclude amyloidosis. **Blood tests** for monoclonal proteins (e.g., serum free light chains, immunofixation) if AL amyloidosis is also a consideration   1. **Heart Failure Management:**   Optimize volume status with careful diuretic use (if there are signs of fluid overload). Cautious use of afterload-reducing agents (ACE inhibitors/ARBs) if blood pressure allows, but be aware that patients with amyloid may be sensitive to hypotension. **Beta-blockers** may be poorly tolerated if there is advanced infiltration, but can be considered at low doses if ischemia or tachyarrhythmias are present   1. **Monitoring & Follow-Up:**   Close clinical follow-up to assess changes in functional capacity (NYHA class). Repeat echocardiogram in **6–12 months** or sooner if symptoms worsen   1. **Lifestyle & Comorbidities:**   Ensure adequate **blood pressure** and **arrhythmia** control if present. Address other risk factors (lipid profile, smoking, diabetes control, etc.) |
| **Conclusion** | This 82-year-old male with dyspnea (NYHA II) and a history of carpal tunnel syndrome demonstrates **marked LV wall thickening, small LV cavity, mildly reduced EF, and significant diastolic dysfunction**, raising suspicion of **cardiac amyloidosis** or another infiltrative cardiomyopathy. Further specialized diagnostic steps are recommended. Careful heart failure management and regular follow-up are crucial to monitor disease progression and address symptoms. |

| **Case** | **Case #19 (HOCM): male, 70 years old, no symptoms** |
| --- | --- |
| **Findings** | 1. **Left Ventricle (LV):**   **IVSd (Interventricular Septal Thickness):** 20 mm, significantly thickened (normal upper limit ~11–12 mm for females, ~12 mm for males), indicating marked LV hypertrophy  **LVEDD (End-Diastolic Diameter):** 46 mm, within normal limits (approx. 42–58 mm for males), albeit on the smaller side of normal  **LVEF (Ejection Fraction):** 60%, Normal systolic function.   1. **Aorta: Ascending Aorta:** 42 mm, slightly above the upper normal threshold (~40 mm), suggesting mild dilation or upper-normal dimension. 2. **Left Atrial Volume Index (LAVI):** 29 ml/m², normal (<34 ml/m²), indicating no significant chronic elevation of left atrial pressure/volume. 3. **Right Ventricle (RV):**   **RV Basal Diameter:** 26 mm, normal (reference <42 mm)  **TAPSE (Tricuspid Annular Plane Systolic Excursion):** 19 mm, normal (≥17 mm indicates preserved RV systolic function)   1. **Valves:**   **Aortic Valve Mean Gradient:** 6 mmHg, normal or near-normal (<10 mmHg), no evidence of significant stenosis  **Other Valvular Gradients (Mitral, etc.):** Not provided, but no high gradients reported   1. **Diastolic Function:**   **E/A Ratio:** 0.64 (reduced, suggestive of impaired relaxation/Grade I diastolic dysfunction), **E/e′:** 3.7 (low/normal, indicating **normal filling pressures** at rest), **Medial e′:** 5.6 cm/s and **Lateral e′:** 7.7 cm/s (slightly reduced or borderline, consistent with mild diastolic impairment but no marked elevation of filling pressure). |
| **Interpretation** | 1. **Marked LV Hypertrophy with normal EF:** The thick interventricular septum (20 mm) combined with a small-to-normal LV cavity suggests concentric hypertrophy. Common causes include **longstanding hypertension** or, less commonly at age 70, **hypertrophic cardiomyopathy** (though that typically presents earlier or has a family history). 2. **Mild Diastolic Dysfunction (Grade I):** A low E/A ratio (<1) is compatible with impaired relaxation, but **normal filling pressures** (E/e′ of 3.7) and a normal-sized left atrium suggest that this diastolic dysfunction has not led to significant chronic elevations in LV filling pressure. 3. **Borderline Mild Ascending Aortic Dilation:** An ascending aorta of 42 mm is slightly above the usual upper normal limit (~40 mm), warranting observation. 4. **Normal Right Ventricular Size and Function:** TAPSE of 19 mm indicates preserved RV systolic performance. |
| **Recommendations** | 1. **Evaluate for Hypertension or Other Causes of LVH**   Ensure blood pressure is well controlled if hypertension is present; consider ambulatory BP monitoring or repeated measurements if uncertain. If there is suspicion of hypertrophic cardiomyopathy (e.g., family history, new murmurs, ECG changes), further evaluation (e.g., cardiac MRI or genetic testing) might be considered.   1. **Lifestyle & Cardiovascular Risk Management**   If hypertensive, aim for guideline-directed BP goals (e.g., <130/80 mmHg if tolerated). Encourage a heart-healthy diet, routine exercise, and adherence to standard cardiovascular risk reduction measures (lipid management, diabetes screening, etc.).   1. **Monitoring**   Periodic echocardiograms (e.g., every 12–24 months) to monitor LV wall thickness, aortic dimension, and diastolic parameters. If the aorta is truly dilated (≥45 mm), consider more frequent imaging to watch for expansion, depending on clinical context.   1. **Clinical Follow-Up**   Even though the patient is currently asymptomatic, watch for signs of heart failure (exercise intolerance, dyspnea) or arrhythmias. If new symptoms develop (e.g., syncope, chest pain, palpitations), reevaluate promptly. |
| **Conclusion** | This 70-year-old asymptomatic male demonstrates **significant LV hypertrophy with normal systolic function**, mild diastolic dysfunction, and mildly enlarged ascending aorta. There is no significant valvular pathology. Further evaluation for hypertension or other causes of LVH is recommended, alongside routine follow-up echocardiography and risk factor management. The overall cardiac function appears compensated, with no immediate interventions required beyond monitoring and possible blood pressure optimization. |

| **Case** | **Case #20 (DCM and aneurysm of ascending aorta): male, 75 years old, no symptoms** |
| --- | --- |
| **Findings** | 1. **Left Ventricle (LV):**   **IVSd (Interventricular Septum):** 11 mm (normal range, indicating no significant hypertrophy)  **LVEDD (Left Ventricular End-Diastolic Diameter):** 65 mm (dilated; normal upper limit ~58 mm for males)  **LVEF (Left Ventricular Ejection Fraction):** 34% (moderately to severely reduced systolic function)   1. **Aorta: Ascending Aorta:** 43 mm (mildly enlarged or upper-normal range) 2. **Left Atrial Volume Index (LAVI):** 35 ml/m² (borderline enlarged; normal <34 ml/m²) 3. **Right Ventricle (RV):**   **RV Basal Diameter:** 20 mm (on the smaller side of normal; typical normal range can be ~25–41 mm)  **TAPSE (Tricuspid Annular Plane Systolic Excursion):** 26 mm (normal or high-normal, indicating preserved or even robust RV systolic function)   1. **Valves:**   **Aortic Valve Mean Gradient:** 5 mmHg (normal, <10 mmHg)  **Mitral Valve Mean Gradient:** Not provided. **No indication of significant stenosis from provided data**  Tricuspid Valve: **TR Gradient:** 26 mmHg (normal; no clear pulmonary hypertension)   1. **Diastolic Function:**   **E/A Ratio:** 1.23 (can be normal or pseudonormal pattern; further evaluation usually needed with tissue Doppler). **E/e′:** 29.1 (significantly elevated, indicating high LV filling pressures). **Medial e′ and Lateral e′:** Both 4.0 cm/s (reduced, consistent with impaired myocardial relaxation).   1. Hemodynamics   **Inferior Vena Cava (IVC):** 17 mm (within normal range, collapsibility unknown) |
| **Interpretation** | 1. **Dilated Left Ventricle with Reduced Ejection Fraction**: LVEDD of 65 mm and LVEF of 34% are consistent with **dilated cardiomyopathy** or significant remodeling, which may be a **post-myocarditis sequela** given the patient’s history. 2. Despite **marked LV dilation** and **reduced systolic function**, the patient is asymptomatic. This may represent a compensated stage of systolic dysfunction. 3. **Elevated Filling Pressures** (E/e′ = 29.1) indicate advanced diastolic dysfunction or high end-diastolic pressures, often seen in heart failure with reduced EF. 4. **Right Ventricular Function** appears preserved (TAPSE 26 mm), although the measured RV basal diameter is relatively small at 20 mm. 5. **Mildly Enlarged Aorta** at 43 mm and borderline **LAVI** at 35 ml/m²; these may not be hemodynamically significant but warrant observation. |
| **Recommendations** | 1. **Heart Failure Management**   Consider standard therapy for HFrEF (Heart Failure with Reduced Ejection Fraction): **ACE Inhibitor (or ARB/ARNi)** to reduce afterload and support remodeling. **Beta-Blocker** (e.g., carvedilol, bisoprolol) if no contraindications, introduced at low dose and titrated up. **Mineralocorticoid Receptor Antagonist (MRA)** if potassium and renal function allow. **Diuretics** (e.g., loop diuretics) for volume management if signs of fluid overload develop.   1. **Follow-Up Echocardiography**   Reassess **LV size, EF, and diastolic parameters** in **3–6 months** to track any changes or improvement, particularly if initiating or optimizing heart failure therapy.   1. **Lifestyle and Risk Factor Optimization**   Ensure **blood pressure** and **lipids** are well controlled. Encourage **exercise** at a level tolerated without exacerbating symptoms. **Smoking cessation** if applicable and moderate alcohol use only.   1. **Additional Investigations**   If there is any suspicion of ongoing myocarditis or ischemic disease, consider **cardiac MRI** for detailed tissue characterization. Evaluate for arrhythmias (e.g., Holter monitoring) given structural heart disease |
| **Conclusion** | This 75-year-old male with a history of myocarditis exhibits **dilated LV geometry and moderately to severely reduced systolic function** (EF 34%), alongside **significantly elevated filling pressures**. He remains asymptomatic, suggesting a compensated state. Recommended management includes guideline-directed medical therapy for HFrEF, follow-up echocardiography to monitor changes, and optimization of cardiovascular risk factors. |

| **Case** | **Case #21 (ICM): male, 58 years old, no symptoms** |
| --- | --- |
| **Findings** | 1. **Left Ventricle (LV):**   **IVSd (Interventricular Septum):** 11 mm (within normal limits, no marked hypertrophy)  **LVEDD (End-Diastolic Diameter):** 64 mm (significantly dilated; normal upper limit ~58 mm for males).  **LVEF (Ejection Fraction):** 27% (severely reduced systolic function):   1. **Aorta: Ascending Aorta:** 37 mm (normal range, <40 mm typically) 2. **Left Atrial Volume Index (LAVI):** 30 ml/m² (upper-normal/borderline; normal <34 ml/m²). 3. **Right Ventricle (RV):**   **RV Basal Diameter:** 38 mm (normal <42 mm)  **TAPSE (Tricuspid Annular Plane Systolic Excursion):** 29 mm (on the higher side of normal or possibly hyperdynamic RV longitudinal function)   1. **Valves:**   **Aortic Valve Mean Gradient:** 5 mmHg (normal, indicating no significant stenosis)  **Mitral Valve Mean Gradient:** 2 mmHg (normal)   1. **Diastolic Function:**   **E/A Ratio:** 0.58 (suggestive of impaired relaxation/Grade I diastolic dysfunction). **E/e':** 16.1 (elevated, indicative of increased LV filling pressures). **Medial e':** 4.6 cm/s and **Lateral e':** 3.3 cm/s (both low, consistent with diastolic dysfunction) |
| **Interpretation** | 1. **Severe Left Ventricular Systolic Dysfunction** with significant LV dilation (LVEDD 64 mm, EF 27%). This is consistent with **ischemic cardiomyopathy** or **dilated cardiomyopathy** in the context of known coronary heart disease. 2. **Diastolic Dysfunction** is also evident (low E/A, low e’ velocities, elevated E/e’), reflecting elevated filling pressures. 3. Despite the severely reduced LV function, the **right ventricle** shows a surprisingly high TAPSE (29 mm), suggesting preserved or even hyperdynamic RV longitudinal function—this could be genuine or reflect measurement variability/technique. 4. **Left Atrial Size** is borderline (LAVI 30 ml/m²), not significantly enlarged despite increased LV filling pressures, which may indicate a subacute or not yet chronically remodeled state. |
| **Recommendations** | 1. **Heart Failure Management (HFrEF)**   **Guideline-Directed Medical Therapy (GDMT)** if not already initiated: **ACE inhibitor (or ARB/ARNi)** to reduce afterload and support remodeling. **Beta-blocker** (carvedilol, bisoprolol, or metoprolol succinate) started at low dose and titrated carefully. **Mineralocorticoid Receptor Antagonist (MRA)** if renal function and potassium levels permit. **Diuretics** (e.g., loop diuretics) for volume management if evidence of fluid overload develops.   1. **Further Cardiac Assessment**   Evaluate for **ischemia/revascularization options** if not already addressed, given known coronary heart disease and severely reduced EF. Consider **ICD (Implantable Cardioverter-Defibrillator)** evaluation if the EF remains <35% after optimal medical therapy (for primary prevention of sudden cardiac death).   1. **Monitoring**   **Repeat Echocardiogram** in ~3–6 months after optimizing therapy to assess for changes in LV size, EF, and filling pressures. **Clinical Follow-Up** to monitor for any heart failure symptoms (dyspnea, fatigue, edema) given the low EF.   1. **Lifestyle & Risk Factor Optimization**   Strict control of **blood pressure**, **lipids**, **glucose** (if applicable). Emphasis on **smoking cessation** (if relevant) and **heart-healthy diet**. Engage in **light-to-moderate exercise** as tolerated, and cardiac rehabilitation if indicated. |
| **Conclusion** | This 58-year-old male with known coronary heart disease demonstrates **severe LV dilation and markedly reduced EF (27%)**, consistent with an **ischemic or dilated cardiomyopathy**. Elevated filling pressures suggest concurrent diastolic dysfunction, while the RV appears to have normal or possibly hyperdynamic function based on TAPSE. **Guideline-directed medical therapy** for HFrEF should be optimized, and follow-up imaging is recommended to monitor progression or improvement of cardiac function. |

**Table S2: LLM-generated report of clinical cases (14-21, Group 2)**

| **Fictional cases**  **(1-13, Group 1)** | **1** | **2** | **3** | **4** | **5** | **6** | **7** | **8** | **9** | **10** | **11** | **12** | **13** |
| --- | --- | --- | --- | --- | --- | --- | --- | --- | --- | --- | --- | --- | --- |
| **Accuracy of findings and interpretation**  **(1-5 points)** | 3 | 5 | 5 | 4 | 4 | 4 | 3 | 5 | 5 | 3 | 3 | 4 | 3 |
| **Appropriateness of recommendations**  **(1 – 3 points)** | 3 | 2 | 3 | 3 | 3 | 3 | 2 | 3 | 3 | 2 | 3 | 2 | 2 |
| **Total**  **(8 points)** | 6 | 7 | 8 | 7 | 7 | 7 | 5 | 8 | 8 | 5 | 6 | 6 | 5 |

| **Clinical cases**  **(14-21, Group 2)** | **14** | **15** | **16** | **17** | **18** | **19** | **20** | **21** |
| --- | --- | --- | --- | --- | --- | --- | --- | --- |
| **Accuracy of findings and interpretation**  **(1-5 points)** | 5 | 5 | 3 | 4 | 3 | 5 | 5 | 5 |
| **Appropriateness of recommendations**  **(1 – 3 points)** | 3 | 3 | 3 | 3 | 3 | 3 | 3 | 3 |
| **Total**  **(8 points)** | 8 | 8 | 6 | 7 | 6 | 8 | 8 | 8 |

**Table S3: Scoring results of all cases provided to the LLM**
